# On the propagation across the big bounce in an open quantum FLRW cosmology

**DOI:** 10.1140/epjc/s10052-022-10874-0

**Published:** 2022-10-13

**Authors:** Emmanuele Battista, Harold C. Steinacker

**Affiliations:** Faculty of Physics, University of Vienna, Boltzmanngasse 5, 1090 Vienna, Austria

## Abstract

The propagation of a scalar field in an open FLRW bounce-type quantum spacetime is examined, which arises within the framework of the IKKT matrix theory. In the first part of the paper, we employ general-relativity tools to study null and timelike geodesics at the classical level. This analysis reveals that massless and massive non-interacting particles can travel across the big bounce. We then exploit quantum-field-theory techniques to evaluate the scalar field propagator. In the late-time regime, we find that it resembles the standard Feynman propagator of flat Minkowski space, whereas for early times it governs the propagation across the big bounce and gives rise to a well-defined correlation between two points on opposite sheets of the spacetime.

## Introduction

Bouncing cosmology has been proposed in the literature either as alternatives or completions of the inflationary paradigm. In Refs. [1–4], it has been shown that the big-bang singularity can be regularized by employing a slight modification of Einstein theory allowing for degenerate metrics. The three-dimensional submanifold of the spacetime where the metric is degenerate represents a spacetime defect and it has been recently suggested that its origin can be explained within the IIB matrix model [5–8]. Universes having a bouncing-like behavior have been addressed in the context of *f*(*R*) theories in Refs. [9–11] and in Gauss–Bonnet modified gravity in Ref. [12]. Furthermore, the authors of Refs. [13–15] have constructed classical nonsingular bouncing models by means of generalized cubic Galileon theories, which permit to realize a pattern where the null energy condition is violated without introducing ghost and gradient instabilities. Another viable theory is represented by the matter bounce scenario [16]. Last, the bounce mechanism has been investigated in the framework of loop quantum cosmology [17–20] and string cosmology [21, 22]. For a review on bouncing cosmologies we refer the reader to Refs. [23–26].

Recently, solutions of matrix models have been found [5–7], which can be interpreted as 3 + 1-dimensional quantum geometries^1^ describing an effective FLRW cosmology with a big bounce (BB). The underlying model is known as IKKT model [33], which has been proposed as a constructive definition of (some corner of) string theory. These solutions or backgrounds have an intrinsic quantum structure, with spacetime uncertainty or “fuzzyness” akin to quantum mechanical phase space. In this framework, a classical spacetime geometry is recovered in the semi-classical or IR regime, while the quantum structure of geometry becomes important only in the UV regime, i.e. at very short distances. In particular, the singularity of classical geometry at the BB is completely under control. Fluctuations of such a background lead to fields, including scalar fields, gauge fields, and gravitons; in fact for the geometry given in [7], a whole tower of higher-spin gauge fields arises, which is described by a ghost-free higher-spin gauge theory [34].

In particular, the framework of matrix models allows to study the physics on such backgrounds with a BB. This study was initiated in [35] on a 1 + 1-dimensional toy model, which allowed to compute the propagator for the global geometry including the BB. Furthermore, the Bogoljubov coefficients which govern the asymptotic properties of fields propagating in and out of the BB were obtained.

In the present paper, we extend the results of [35] to the case of 3+1 dimensions. We obtain explicitly the fluctuation modes of a scalar field on the 3 + 1-dimensional FLRW background in the semi-classical regime, paying special attention to the asymptotic regimes of late times and close to the BB. This then allows to compute the propagator explicitly, using a path integral approach provided by the underlying matrix model framework. Remarkably, the propagator is found to be regular at the BB, and has the local structure of a Feynman propagator at late times. This means that physical modes can propagate across the BB singularity in a well-defined way, and some of their structure will survive.

The main message of this paper is that matrix models provide a suitable framework for quantum geometry, which allows to address and resolve the singularities which arise in the framework of general relativity. Moreover, the present example demonstrates how a time evolution and a 3 + 1-dimensional causal structure can emerge from the underlying matrix model, which has no a priori notions of space and time. It should be emphasized that the dynamics is governed here by the matrix model, which is different from general relativity, at least at the classical level. As demonstrated in [36], the Einstein–Hilbert action does arise upon including 1-loop effects in the IKKT model, under suitable assumptions. This will of course affect some of the results of the present paper, however we expect that the qualitative features of the present classical analysis will also apply after including quantum effects.

The paper is morally divided into two parts, a classical and a quantum one. The two parts are consistent with each other. In the classical part, we study some aspects of the present FLRW geometry, with special emphasis on the near-BB regime. In particular, we elaborate the geodesics, and show that they extend smoothly across the BB. The BB singularity is found to be rather “mild” in a sense that will be explained below. The propagator is obtained in the quantum setting, by computing explicitly the (free) path integral of modes as defined by the matrix model. At late times, we recover again the standard Feynman propagator with the appropriate $i\varepsilon$ structure. At early times near the BB, the propagator also turns out to be well-defined, and allows to study the propagation of scalar particles across the BB. This result agrees with the classical analysis regarding null and timelike geodesic.

*Notations.* We use metric signature $(-,+,+,+)$. Greek indices take values 0, 1, 2, 3. The flat metric is indicated by $\eta ^{\alpha \beta }=\eta _{\alpha \beta }=\mathrm{diag}(-1,1,1,1)$.

## The background geometry

We recall [7] that the background $\mathcal{M}^{3,1}$ under consideration can be described semi-classically as a projection of fuzzy $H^4_n$, which is obtained from five matrices $X^a \sim x^a$ interpreted as quantizations of five embedding functions

1 $$\begin{aligned} x^a: H^4 \hookrightarrow {\mathbb R}^{4,1} \end{aligned}$$

where $a=0,\ldots ,4$. A convenient parametrization of this four-dimensional hyperboloid is as follows

2 $$\begin{aligned} \begin{bmatrix} x^0 \\ x^1 \\ x^2 \\ x^3 \\ x^4 \end{bmatrix} = R \begin{bmatrix} \cosh (\eta ) \begin{pmatrix} \cosh (\chi ) \\ \sinh (\chi )\sin (\theta ) \cos (\varphi ) \\ \sinh (\chi )\sin (\theta ) \sin (\varphi ) \\ \sinh (\chi )\cos (\theta ) \end{pmatrix} \\ \sinh (\eta ) \end{bmatrix}, \ \end{aligned}$$

for $\eta \in {\mathbb R}$. Note that $\chi$ can be restricted to be positive. Projecting this along the $x^4$ axis leads to a 2-sheeted cover of the following region

3 $$\begin{aligned} x_\mu x^\mu \leqslant -R^2, \end{aligned}$$

where the upper sheet (“post-BB”, corresponding to $x^4>0$) is covered by $\eta > 0$, while the lower sheet (“pre-BB”, corresponding to $x^4<0$) is covered by $\eta < 0$. The BB separates these sheets, and corresponds to $x_\mu x^\mu = -R^2$. This leads to the following parametrization of $\mathcal{M}^{3,1}$

4 $$\begin{aligned} \begin{pmatrix} x^0 \\ x^1 \\ x^2 \\ x^3 \end{pmatrix} = R \cosh (\eta ) \begin{pmatrix} \cosh (\chi ) \\ \sinh (\chi )\sin (\theta ) \cos (\varphi ) \\ \sinh (\chi )\sin (\theta ) \sin (\varphi ) \\ \sinh (\chi )\cos (\theta ) \end{pmatrix} . \end{aligned}$$

Note that the flow of time will be along increasing $\eta$ on both sheets; this arises from the $i\varepsilon$ regularization discussed in Sect. 5.

In principle, we can restrict $\chi$ to be either positive or negative on either sheet. However, we will see in Sects. 4 and 5 that it is convenient to choose $\chi >0$.

### Effective metric

To understand the effective metric on $\mathcal{M}^{3,1}$, we recall [7] that the background solution $T^\mu$ of the matrix model leads to the following kinetic term

5 $$\begin{aligned} S[\phi ] = - \mathrm {Tr}[T^\mu ,\phi ][T_\mu ,\phi ] \end{aligned}$$

which governs *all* fluctuations in the matrix model. Using the semi-classical relation $[T^\mu ,.] \sim i\{t^\mu ,.\}$ in terms of Poisson brackets (here $t^\mu$ represents the semiclassical limit of $T^\mu$) and recalling $\{t^\mu ,x^\nu \} = \sinh (\eta )\delta ^\mu _\nu$, this can be rewritten uniquely in the standard form [7, 37]

6 $$\begin{aligned} S[\phi ] = - \mathrm {Tr}[T^\mu ,\phi ][T_\mu ,\phi ] \sim \int d^4 x\,\sqrt{|G|}G^{\mu \nu }\partial _\mu \phi \partial _\nu \phi \ \end{aligned}$$

where [7]

7 $$\begin{aligned} G^{\mu \nu }&= \vert \sinh (\eta ) \vert ^{-3}\, \gamma ^{\mu \nu }, \quad \gamma ^{\alpha \beta } = \sinh ^2(\eta ) \eta ^{\alpha \beta }, \end{aligned}$$

dropping some irrelevant constant (here $\gamma ^{\mu \nu }$ is an auxiliary metric which is relevant for the torsion). This metric can be recognized as *SO*(3, 1)-invariant FLRW metric,

8 $$\begin{aligned} d s^2_G = G_{\mu \nu } d x^\mu d x^\nu&= -R^2 \vert \sinh (\eta ) \vert ^3 d \eta ^2 \nonumber \\&\quad + R^2 \vert \sinh (\eta )\vert \cosh ^2(\eta )\, d \Sigma ^2 \ \nonumber \\&= -d t^2 + a^2(t)d\Sigma ^2 \, . \end{aligned}$$

where

9 $$\begin{aligned} d\Sigma ^2 = d\chi ^2 + \sinh ^2\chi (d\theta ^2 + \sin ^2 \theta d\varphi ^2), \end{aligned}$$

is the invariant length element on the space-like hyperboloids $H^3$ (with $-\infty \leqslant \chi <\infty$, $0 \leqslant \theta < \pi$, $0 \leqslant \varphi <2\pi$). Equivalently, we can write

10 $$\begin{aligned} d\Sigma ^2=\dfrac{dr^2}{1+r^2} + r^2 (d\theta ^2 + \sin ^2 \theta d\varphi ^2), \end{aligned}$$

with

11 $$\begin{aligned} r = \sinh \chi . \end{aligned}$$

From Eq. (8), we can read off the cosmic scale parameter $a(\eta )$ and the relation linking the differentials *dt* and $d\eta$, i.e.,

12 $$\begin{aligned} \vert a(\eta ) \vert&= R \cosh (\eta ) \vert \sinh (\eta )\vert ^{1/2} , \end{aligned}$$

13 $$\begin{aligned} d t&= R \vert \sinh (\eta )\vert ^{3/2} d\eta . \end{aligned}$$

For late times, we have

14 $$\begin{aligned} a^2(t) \ {\mathop {\sim }\limits ^{t\rightarrow \infty }} \ R^2\vert \sinh (\eta )\vert ^3 . \end{aligned}$$

This is a reasonable FLRW cosmology given the simplicity of the model, which is asymptotically coasting at late time with $a(t) \sim \frac{3}{2} t$, cf. Refs. [38, 39]. Note that it arises directly from the matrix model, without using or assuming general relativity. Last, it is worth mentioning that the scale factor (12) can be either odd or even (the odd solution, in particular, may be of interest as discussed in Ref. [40]).

## Classical analysis of the FLRW spacetime

In this section, we perform a classical investigation of the FLRW geometry (8). Curvature invariants are considered in Sect. 3.1, whereas null and timelike geodesics are studied in Sect. 3.2. We conclude the section with the analysis of some cosmological observables (see Sect. 3.3).

### Curvature invariants

In order to describe the behaviour of the spacetime near the BB, we begin our analysis with the investigation of some curvature invariants. Starting from the metric (8), we find that the Kretschmann scalar is

15 $$\begin{aligned} R_{\mu \nu \rho \sigma }R^{\mu \nu \rho \sigma }= & {} \dfrac{3}{32R^4 \sinh ^{10}\left( \eta \right) } \left[ 171-60 \cosh \left( 2 \eta \right) \right. \nonumber \\&\left. +25\cosh \left( 4 \eta \right) \right] , \end{aligned}$$

the squared Ricci tensor reads as

16 $$\begin{aligned} R_{\mu \nu }R^{\mu \nu }&=\dfrac{3}{512R^4 \sinh ^{10}\left( \eta \right) \cosh ^{4}\left( \eta \right) }\nonumber \\&\quad \times \Bigl [1635-488 \cosh \left( 6\eta \right) +97 \cosh \left( 8\eta \right) \nonumber \\&\quad -384 \sinh ^4\left( \eta \right) +4 \cosh \left( 4\eta \right) \left( 287-288\sinh ^4\left( \eta \right) \right) \nonumber \\&\quad +8\cosh \left( 2\eta \right) \left( 320\sinh ^4\left( \eta \right) -91\right) \Bigr ], \end{aligned}$$

whereas the (topological) Euler invariant is [41–43]

17 $$\begin{aligned}&{}^{\star }R^{\star }_{\mu \nu \rho \sigma }R^{\mu \nu \rho \sigma }= \dfrac{3}{4R^4 \sinh ^{10}\left( \eta \right) \cosh ^{2}\left( \eta \right) }\nonumber \\&\quad \times \left[ 11-12\cosh \left( 2\eta \right) +9\cosh \left( 4\eta \right) -32 \sinh ^4\left( \eta \right) \right] , \end{aligned}$$

the star indicating the duality operation. It is clear that the scalars (15)–(17) blow up at the BB, i.e., at $\eta =0$.

### Null and timelike geodesics

In this section, we investigate null and timelike geodesics of the FLRW geometry having $\theta$ and $\varphi$ constant.

Null geodesics can be described in terms of the function $\chi (\eta )$. We seek a solution which reaches the BB at $\eta =0$ after having travelled toward it for $\eta <0$ and away from it when $\eta >0$. Therefore, it follows from Eq. (8) that the motion in the outward $\chi$-direction is parametrized by the differential equation

18 $$\begin{aligned} \dfrac{d \chi }{d \eta } = \vert \tanh \eta \vert , \end{aligned}$$

which, with the boundary condition $\chi (\eta =0)=0$, leads to

19 $$\begin{aligned} \chi (\eta ) = \left\{ \begin{array}{ll} \log \left( \cosh \eta \right) , &{} \eta \geqslant 0,\\ -\log \left( \cosh \eta \right) , &{} \eta <0. \end{array} \right. \end{aligned}$$

**Fig. 1:**
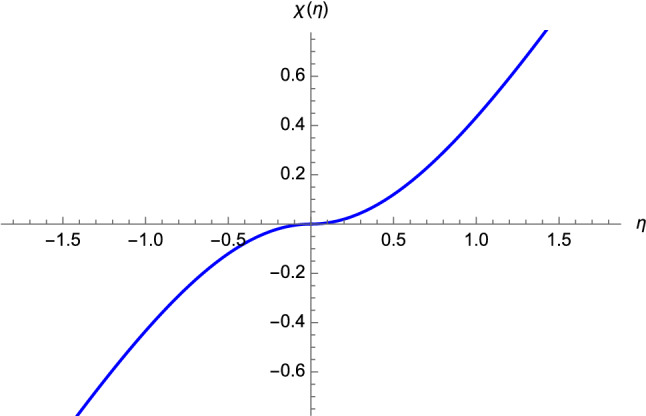
Null geodesic motion having $\theta$ and $\varphi$ constants (cf. Eq. (19)). It is clear that the function $\chi (\eta )$ is continuous at the BB

**Fig. 2:**
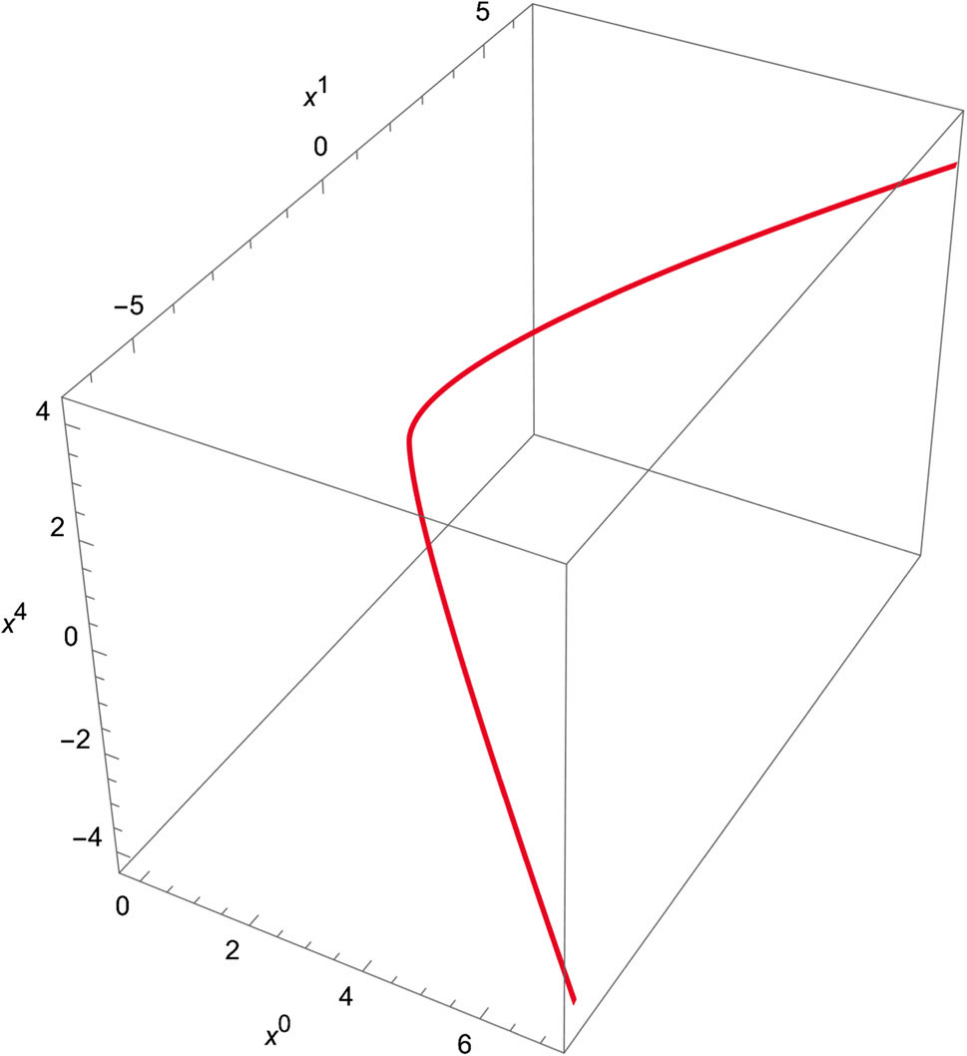
Null geodesic motion obtained by means of Eqs. (2) and (19). The following values have been chosen: $R=1$, $\theta =\pi /2$, and $\varphi =0$

The behaviour of the solution (19) is shown in Fig. 1, whereas Fig. 2 represents the plot obtained by means of the embedding functions (2).

Having obtained a continuous geodesic solution which can be extended uniquely at $\eta =0$, we can conclude that light (and hence the physical information) can travel across the BB, despite the singularity occurring in the invariants (15)–(17).

It is interesting to work out the solution $\chi = \chi (t)$ of null geodesics (with $\theta$ and $\varphi$ constant) for early times, i.e., when $t \rightarrow t_0$ (with $t_0 \in \mathbb {R}$) or, equivalently $\eta \rightarrow 0$. First of all, from Eq. (13) jointly with the condition $t(\eta =0)=t_0$, we obtain

20 $$\begin{aligned} t(\eta ) -t_0&\; \overset{\eta \rightarrow 0}{\sim } \; \dfrac{2}{5} R \eta \vert \eta \vert ^{3/2} =\left\{ \begin{array}{ll} \dfrac{2}{5} R \eta ^{5/2}, &{} \eta \geqslant 0,\\ \dfrac{2}{5} R \eta \left( -\eta \right) ^{3/2}, &{} \eta <0. \end{array} \right. \end{aligned}$$

The inversion of the above function yields

21 $$\begin{aligned} \eta (t)&\; \overset{t \rightarrow t_0}{\sim } \left\{ \begin{array}{ll} \left( \dfrac{5}{2R}\right) ^{2/5} \left( t-t_0\right) ^{2/5}, &{} t \geqslant t_0,\\ -\left( \dfrac{5}{2R}\right) ^{2/5} \left( t-t_0\right) ^{2/5}, &{} t<t_0, \end{array} \right. \end{aligned}$$

where we note that $\eta (t=t_0)=0$.

**Fig. 3:**
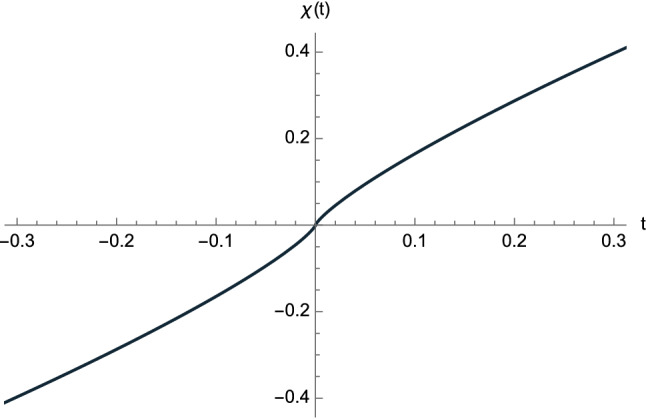
The function (24) describing early-time null geodesic having $\theta$ and $\varphi$ constant. We have chosen $R=1$ and $t_0=0$

We are now ready to obtain the expression of the cosmic scale factor valid near the BB. Indeed, by means of Eqs. (12) and (21), we have

22 $$\begin{aligned} a(t)&\; \overset{t \rightarrow t_0}{\sim } \left\{ \begin{array}{ll} R\left( \dfrac{5}{2R}\right) ^{1/5} \vert t-t_0 \vert ^{1/5}, &{} t \geqslant t_0,\\ -R\left( \dfrac{5}{2R}\right) ^{1/5} \vert t-t_0 \vert ^{1/5}, &{} t<t_0, \end{array}\right. =R\left( \dfrac{5}{2R}\right) ^{1/5} ( t-t_0 )^{1/5}, \end{aligned}$$

which vanishes at $t=t_0$. We note that *a*(*t*) is positive (resp. negative) for $t>t_0$ (resp. $t<t_0$). The sign of *a*(*t*) drops out in the metric (8), but the above choice has no cusp. The equation of null geodesics (with $\theta$ and $\varphi$ constant) can be written in the equivalent form

23 $$\begin{aligned} \dfrac{d \chi }{d t} = \dfrac{1}{\vert a(t)\vert }, \end{aligned}$$

which in view of (22) gives the desired early-time solution

24 $$\begin{aligned} \chi (t)&\; \overset{t \rightarrow t_0}{\sim } \left\{ \begin{array}{rl} \dfrac{1}{2}\left( \dfrac{5}{2R}\right) ^{4/5} \left( t-t_0 \right) ^{4/5}, &{} t \geqslant t_0,\\ -\dfrac{1}{2}\left( \dfrac{5}{2R}\right) ^{4/5} \left( t-t_0 \right) ^{4/5}, &{} t<t_0, \end{array} \right. \end{aligned}$$

see Fig. 3. The above solution can also be obtained if we first expand Eq. (19) about $\eta = 0$, and then exploit Eq. (21).

At this stage, we analyze the timelike geodesics of a non-comoving observer. Hence, let

25 $$\begin{aligned} u^\alpha = \dfrac{dx^\alpha (\tau )}{d \tau }, \end{aligned}$$

denote the unit timelike four-velocity vector of such observer, whose proper time is indicated with $\tau$. If we suppose, like before, that the observer moves along the direction of constant $\theta$ and $\varphi$, then from the normalization condition

26 $$\begin{aligned} g_{\alpha \beta } u^\alpha u^\beta = -1, \end{aligned}$$

we obtain

27 $$\begin{aligned} R^2 \vert \sinh \eta \vert ^3 \left( \dfrac{d \eta (\tau )}{d\tau }\right) ^2= 1+a^2(\eta )\left( \dfrac{d \chi (\tau )}{d\tau }\right) ^2. \end{aligned}$$

Furthermore, if we consider the $\chi$-translational Killing vector $\xi ^\alpha$, the conserved momentum $\Pi$ of the non-comoving observer is given by

28 $$\begin{aligned} \Pi = \xi ^\alpha u_\alpha = a^2(\eta )\dfrac{d \chi (\tau )}{d\tau }. \end{aligned}$$

The above equation gives a relation to express $d \chi (\tau )/d\tau$ in terms of $\Pi$ and $a(\eta )$, which can be exploited in Eq. (27). In this way, after some manipulations, we end up with the geodesic equation in terms of the function $\chi (\eta )$, i.e.,

29 $$\begin{aligned} \dfrac{d \chi (\eta )}{d\eta }&= \dfrac{d\chi (\tau )/d\tau }{d\eta (\tau )/d\tau }=\dfrac{\vert \tanh \eta \vert }{\sqrt{1+a^2(\eta )/\Pi ^2}}. \end{aligned}$$

**Fig. 4:**
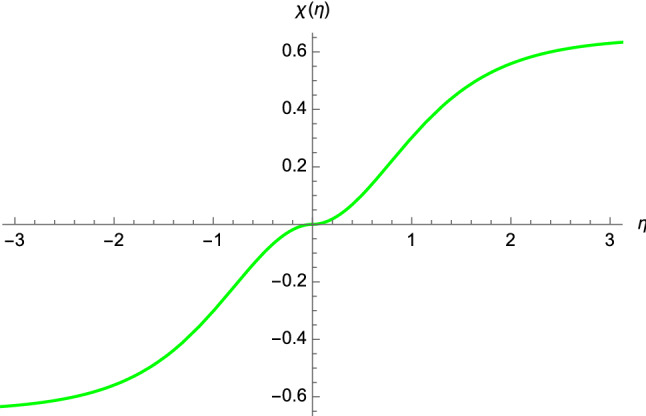
Timelike geodesic of the non-comoving observer obtained by solving numerically Eq. (29) with the boundary condition $\chi (\eta =0)=0$. The following constants have been chosen: $R=1$ and $\Pi =1$

We have solved numerically Eq. (29) with the boundary condition $\chi (\eta =0)=0$. As it is clear from Fig. 4, the non-comoving observer can travel across the BB, likewise a massless particle.

We can provide the analytic solution for the timelike geodesic of the non-comoving observer moving in the $\chi$-direction in the regime of small times. First of all, it is easy to see that the equation of the timelike geodesic of the non-comoving observer in terms of the cosmic time variable is

30 $$\begin{aligned} \dfrac{d\chi (t)}{dt}=\dfrac{1}{\vert a(t)\vert }\dfrac{1}{\sqrt{1+a^2(t)/\Pi ^2}}. \end{aligned}$$

Bearing in mind Eq. (22) and considering, for simplicity, $t_0=0$, $R=1$, and $\Pi =1$, the solution of Eq. (30) with the boundary condition $\chi (t=0)=0$ reads as

31 $$\begin{aligned} \chi (t)&\; \overset{t \rightarrow 0}{\sim } \left\{ \begin{array}{ll} \dfrac{5}{3}\left[ 2-2\sqrt{1+t^{2/5}}+t^{2/5}\sqrt{1+t^{2/5}}\right] , &{} t \geqslant 0,\\ -\dfrac{5}{3}\left[ 2-2\sqrt{1+t^{2/5}}+t^{2/5}\sqrt{1+t^{2/5}}\right] ,&{} t<0. \end{array} \right. \end{aligned}$$

**Fig. 5:**
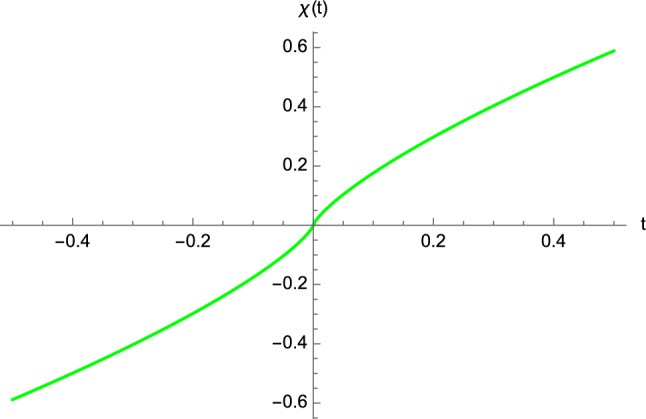
The function (31) describing the early-time timelike geodesic of the non-comoving observer with $t_0=0$, $R=1$, and $\Pi =1$ and the boundary condition $\chi (t=0)=0$

The plot of the function (31) is given in Fig. 5.

Although we have shown that both null and timelike geodesics have no pathological behaviour, it should be noted that the corresponding velocities blow up at the BB, as it is clear from Eqs. (23) and (30).

#### Singularity or not? “Mild singularity” or a new kind of singularity

In the general relativity framework, singularity theorems are based on the criterion that timelike and null geodesic completeness are minimum conditions for a spacetime to be considered singularity-free [44–46]. However, these theorems do not prove that singularities of spacetime are necessarily related to unboundedly large curvature. Indeed, the characterization of singularities via the divergent behaviour of the curvature can be inadequate in some situations (e.g., the case of conical singularity).

Our model is not framed in general relativity, but emerges in the semiclassical limit of the matrix theory. In other words, the cosmic scale factor occurring in the metric (8) is not a solution of Einstein field equations. In our analysis, $a(\eta )$ vanishes at the BB, where, in addition, the invariants (15)–(17) blow up. Furthermore, the effective metric (8) is zero (and hence is degenerate) at $\eta =0$. Despite that, no pathological behaviour occur in the analysis of null and timelike geodesics, as we have shown before. Therefore, if we are to make a comparison with the recipes of general relativity, we could say that our FLRW solution features a “mild singularity”^2^ which does not prevent both massless particles and non-comoving observers from crossing the BB, but introduces some diverging curvature scalars. Moreover, owing to the degenerate character of the metric (8), another option is possible, where we could conclude that we are dealing with a new kind of singularity, which, in principle, might be present also in general relativity, as discussed by Hawking in Ref. [49].

### Cosmological observables

In this section, we evaluate some cosmological observables of our FLRW geometry. In Sect. 3.3.1 we consider the particle horizon, while in Sect. 3.3.2 we deal with the luminosity distance.

#### The particle horizon

In terms of the time coordinate, the particle horizon $\mathscr {D}_{\mathrm{hor}}(t)$ at $t >0$ reads as [50]

32 $$\begin{aligned} \mathscr {D}_{\mathrm{hor}}(t)= a(t) \lim _{t^\star \rightarrow + \infty } \int ^{t}_{-t^\star } \dfrac{dt^\prime }{\vert a(t^\prime )\vert }, \end{aligned}$$

where we note that, for the background under consideration, we are free to choose $-t^\star$ arbitrarily negative. In general relativity settings, this operation would be correct only if the spacetime were singularity-free. However, following our discussion of Sect. 3.2.1, it makes sense to consider such a limit in our model, since we are dealing with a new kind of singularity which cannot be totally explained with the standard tools of Einstein theory.

Bearing in mind Eqs. (12) and (13), the particle horizon $\mathscr {D}_{\mathrm{hor}}(\eta )$ at $\eta >0$ is

33 $$\begin{aligned} \mathscr {D}_{\mathrm{hor}}(\eta )&= a(\eta ) \lim _{\eta ^\star \rightarrow + \infty } \int ^{\eta }_{-\eta ^\star } d\eta ^\prime \vert \tanh \eta ^\prime \vert \nonumber \\&= \left( R \cosh \eta \sqrt{\sinh \eta }\right) \lim _{\eta ^\star \rightarrow + \infty } \left[ \log \left( \cosh \eta ^\star \right) \right. \nonumber \\&\quad \left. +\log \left( \cosh \eta \right) \right] = + \infty , \end{aligned}$$

where $\eta ^\star \equiv \eta (t^\star )$. Since $\mathscr {D}_\mathrm{hor}(\eta )$ diverges, it is possible to receive signals from any coming particle of the spacetime. This can be interpreted as a hint that our model has no need for inflation.

#### Luminosity distance

Let us consider a light signal emitted by a comoving source at time $t_e>0$ which is then detected by a comoving observer at time $t_0>t_e$. If we further suppose that the signal moves keeping $\theta$ and $\varphi$ constant, the luminosity distance $d_L(t_e;t_0)$ is given by [50]

34 $$\begin{aligned} d_L(t_e;t_0)&= r_e \dfrac{a^2(t_0)}{a(t_e)}, \end{aligned}$$

where the coordinate distance $r_e$ travelled by the signal is expressed by the relation

35 $$\begin{aligned} \int _{t_e}^{t_0}\dfrac{dt}{a(t)} = \int _{0}^{r_e}\dfrac{dr}{\sqrt{1+r^2}}= \mathrm{arcsinh \,}r_e. \end{aligned}$$

In our model, it follows from Eqs. (11)–(13) that the luminosity distance can be expressed as

36 $$\begin{aligned} d_L(\eta _e;\eta _0)&= \dfrac{a^2(\eta _0)}{a(\eta _e)}\sinh \left[ \int ^{\eta _0}_{\eta _e} d \eta \tanh \eta \right] \nonumber \\&=\dfrac{a^2(\eta _0)}{a(\eta _e)} \sinh \left[ \log \left( \cosh \eta _0\right) -\log \left( \cosh \eta _e\right) \right] , \end{aligned}$$

where $\eta _e \equiv \eta (t_e)>0$ and $\eta _0 \equiv \eta (t_0)>\eta _e$. It is easy to see that

37 $$\begin{aligned} \lim _{\eta _e \rightarrow 0^{+}} d_L(\eta _e;\eta _0) = + \infty , \end{aligned}$$

which represents an expected result since the cosmic scale factor $a(\eta )$ vanishes at the BB.

## The scalar modes on $\mathcal{M}^{3,1}$

In this second part of the paper, we would like to compute the propagator for a scalar field on the above background, and see if there are any interesting effects due to the BB. A priori it is not evident how to define the propagator, due to the boundary provided by the BB. We take as starting point the definition as 2-point function defined by a Gaussian integral in the matrix model (or rather its semi-classical limit). Schematically,

38 $$\begin{aligned} \langle \phi (x) \phi (y)\rangle = \int dk \, \langle \phi _k(x) \phi _k(y)\rangle , \end{aligned}$$

where

39 $$\begin{aligned} \langle \phi _k(x) \phi _k(y)\rangle = \frac{1}{Z} \int d\phi \, \phi _k(x) \phi _k(y) e^{i S[\phi _k]}, \end{aligned}$$

*Z* being the generating functional. The necessary details for this computation will be provided below.

### Relevant Operators

In this section, we introduce some relevant wave operators which will play a crucial role in our forthcoming analysis.

#### The effective d’Alembertian $\Box _G$

The metric (8) is encoded in the “matrix” d’Alembertian

40 $$\begin{aligned} \Box \ = [T^\mu ,[T_\mu ,.]] \sim \ -\{t^\mu ,\{t_\mu ,.\}\}, \end{aligned}$$

which governs the propagation of a scalar fields $\phi$, and is related to the metric d’Alembertian through

41a $$\begin{aligned} \Box \&\sim |\sinh ^{3} \eta | \Box _G, \end{aligned}$$

41b $$\begin{aligned} \Box _G&= -\frac{1}{\sqrt{|G|}}\partial _\mu \big (\sqrt{|G|}\, G^{\mu \nu }\partial _\nu \big ). \end{aligned}$$

This can be seen by rewriting the action (6) as follows

42 $$\begin{aligned} S[\phi ] = - \int \Omega \phi \{T^\mu \{T_\mu ,\phi \}\} = -\int d^4 x\,\sqrt{|G|} \phi \Box _G\phi \ \end{aligned}$$

where

43 $$\begin{aligned} \Omega&= \frac{1}{\vert \sinh (\eta )\vert } d^4 x = \cosh ^3(\eta ) d\eta \sinh ^2(\chi ) d\chi \sin (\theta )d\theta d\varphi \end{aligned}$$

is the *SO*(4, 1)-invariant volume form on $H^4$ in Cartesian and hyperbolic coordinates (4), respectively (hereafter, we suppose that $\chi >0$). Explicitly, one finds

44 $$\begin{aligned} \Box _G \phi&= \frac{1}{R^2 \vert \sinh ^3 \eta \vert \cosh ^3\eta } \partial _\eta \big (\cosh ^3(\eta )\partial _\eta \phi \big ) \nonumber \\&\qquad + \frac{1}{\vert \sinh \eta \vert }\Delta ^{(3)}_\eta \phi \nonumber \\&\quad =\dfrac{1}{R^2} \left( \dfrac{3}{\sinh \eta \vert \sinh \eta \vert \cosh \eta } \partial _\eta +\dfrac{1}{\vert \sinh \eta \vert ^3 }\partial ^2_\eta \right) \phi \nonumber \\&\qquad + \frac{1}{\vert \sinh \eta \vert }\Delta ^{(3)}_\eta \phi , \end{aligned}$$

(cf. (2.32) in [51]) where $\Delta ^{(3)}_\eta$ is the Laplacian on the space-like three-dimensional hyperboloid $H^3$

45 $$\begin{aligned} \Delta ^{(3)}_\eta \phi&= - \frac{1}{R^2\cosh ^2\eta } \Biggl [ \left( \dfrac{2}{\tanh \chi }\partial _\chi + \partial ^2_\chi \right) \nonumber \\&\quad + \dfrac{1}{\sinh ^2 \chi } \left( \dfrac{1}{\tan \theta } \partial _\theta + \partial ^2_\theta + \dfrac{1}{\sin ^2 \theta }\partial ^2_\varphi \right) \Biggr ]\phi \nonumber \\&\equiv - \frac{1}{R^2\cosh ^2\eta } \Delta ^{(3)}\phi , \end{aligned}$$

where we have defined

46 $$\begin{aligned} \Delta ^{(3)} \phi&\equiv \Biggl [ \left( \dfrac{2}{\tanh \chi }\partial _\chi + \partial ^2_\chi \right) + \dfrac{1}{\sinh ^2 \chi } \nonumber \\&\quad \times \left( \dfrac{1}{\tan \theta } \partial _\theta + \partial ^2_\theta + \dfrac{1}{\sin ^2 \theta }\partial ^2_\varphi \right) \Biggr ] \phi . \end{aligned}$$

We note that to derive Eq. (45) we have exploited that the metric on the space-like $H^3$ is

47 $$\begin{aligned} ds^2\vert _{H^3}= R^2 \cosh ^2 (\eta ) d\Sigma ^2. \end{aligned}$$

#### The Laplacian operator $\Delta _{\mathscr {G}}$ on $H^4$

The induced metric on the four-dimensional hyperboloid $H^4$ is (cf. Eq. (2))

48 $$\begin{aligned} ds^2\vert _{H^4}= \mathscr {G}_{\mu \nu }dx^\mu dx^\nu = R^2 d\eta ^2 + R^2 \cosh ^2 (\eta ) d\Sigma ^2, \end{aligned}$$

where the length element $d\Sigma ^2$ on a spatial standard three-dimensional hyperboloid $H^3$ has been given in Eq. (9). By means of the metric (48), the Laplacian operator $\Delta _{\mathscr {G}}$ on a generic function $\phi =\phi (\eta ,\chi ,\theta ,\varphi )$ reads as

49 $$\begin{aligned} \Delta _{\mathscr {G}} \phi&= \dfrac{1}{\sqrt{\mathscr {G}}}\partial _\mu \left( \sqrt{\mathscr {G}}\mathscr {G}^{\mu \nu } \partial _\nu \phi \right) \nonumber \\&=\dfrac{1}{R^2}\Biggl \{ 3 \tanh (\eta ) \partial _\eta + \partial ^2_\eta +\dfrac{1}{\cosh ^2 (\eta )} \Biggl [ \dfrac{2}{\tanh (\chi )}\partial _\chi +\partial ^2_\chi \nonumber \\&\quad +\dfrac{1}{\sinh ^2(\chi )} \left( \dfrac{1}{\tan (\theta )}\partial _\theta +\partial ^2_\theta +\dfrac{1}{\sin ^2(\theta ) } \partial ^2_\varphi \right) \Biggr ]\Biggr \}\phi , \end{aligned}$$

where $$\sqrt{\mathscr {G}}=R^4 \cosh ^3 (\eta ) \sinh ^2 (\chi ) \sin (\theta )$$.

#### Relations between $\Delta _{\mathscr {G}}$ and $\Delta ^{(3)}_\eta$ and $\Box _G$ and $\Delta ^{(3)}_\eta$

The Laplacian $\Delta _{\mathscr {G}}$ on the four-dimensional hyperboloid $H^4$ and the Laplacian $\Delta ^{(3)}_\eta$ on the spacelike three-dimensional hyperboloid $H^3$ are related by (cf. Eqs. (45) and (49))

50 $$\begin{aligned} \Delta _{\mathscr {G}} = \dfrac{1}{R^2} \left( 3 \tanh (\eta ) \partial _\eta + \partial ^2_\eta \right) - \Delta ^{(3)}_\eta . \end{aligned}$$

On the other hand, the effective d’Alembertian $\Box _G$ is related to $\Delta ^{(3)}_\eta$ as (cf. Eq. (44))

51 $$\begin{aligned} \vert \sinh ^3 \eta \vert \Box _G = \dfrac{1}{R^2} \left( 3 \tanh (\eta ) \partial _\eta + \partial ^2_\eta \right) +\left( \sinh ^2 \eta \right) \Delta ^{(3)}_\eta . \end{aligned}$$

Last, we observe that the effective d’Alembertian $\Box _G$ is related to the Laplacian $\Delta _\mathscr {G}$ on $H^4$ as follows (cf. Eqs. (44) and (49))

52 $$\begin{aligned} R^2 \vert \sinh ^3 \eta \vert \Box _G&= R^2 \Delta _\mathscr {G} - \left( \dfrac{2}{\tanh \chi }\partial _\chi + \partial ^2_\chi \right) \nonumber \\&\quad -\dfrac{1}{\sinh ^2 \chi }\left( \dfrac{1}{\tan \theta }\partial _\theta + \partial _\theta ^2 + \dfrac{1}{\sin ^2 \theta }\partial ^2_\varphi \right) . \end{aligned}$$

An inspection of Eqs. (50)–(52) reveals that the eigenfunctions of the operators $\Delta _{\mathscr {G}}$, $\Delta ^{(3)}$, and $\Box$ share the same building blocks. The eigenfunctions of d’Alembertian operator $\Box$ are derived in the next section, while those of $\Delta _{\mathscr {G}}$ and $\Delta ^{(3)}$ can be found in Appendix A.

### Eigenfunctions of the d’Alembertian operator $\Box$

It is convenient to work out the eigenfunctions of the operator $\Box$ instead of $\Box _G$ for at least two reasons. First of all, the d’Alembertian $\Box$ is self-adjoint with respect to the symplectic volume form (43); secondly, the operator $\Box$ admits handier eigenfunctions than $\Box _G$.

Bearing in mind Eqs. (41a), (44), and (45), the d’Alembertian operator $\Box$ reads as

53 $$\begin{aligned} \Box \phi&= \dfrac{1}{R^2}\Biggl [ 3 \tanh (\eta ) \partial _\eta + \partial ^2_\eta -\tanh ^2 \eta \left( \dfrac{2}{\tanh \chi }\partial _\chi +\partial ^2_\chi \right) \nonumber \\&\quad -\dfrac{\tanh ^2 \eta }{\sinh ^2 \chi } \left( \dfrac{1}{\tan \theta }\partial _\theta +\partial ^2_\theta +\dfrac{1}{\sin ^2 \theta } \partial ^2_\varphi \right) \Biggr ]\phi . \end{aligned}$$

The eigenfunctions of the d’Alembertian operator are defined by the equation

54 $$\begin{aligned} \Box \phi = \lambda \phi , \end{aligned}$$

whose resolution can be tackled via the separation *ansatz*

55a $$\begin{aligned} \phi (\eta ,\chi ,\theta ,\varphi )&= \tilde{\phi }(\eta ,\chi )Y^m_l(\theta ,\varphi ), \end{aligned}$$

55b $$\begin{aligned} \tilde{\phi }(\eta ,\chi )&=f(\eta )g(\chi ), \end{aligned}$$

where the spherical harmonic functions $Y^m_l(\theta ,\varphi )$ of degree *l* and order *m* (with $l \geqslant \vert m \vert$) satisfy the well-known property [52]

56 $$\begin{aligned} \left( \dfrac{1}{\tan \theta }\partial _\theta +\partial ^2_\theta +\dfrac{1}{\sin ^2\theta } \partial ^2_\varphi \right) Y^m_l(\theta ,\varphi )=-l(l+1)Y^m_l(\theta ,\varphi ). \end{aligned}$$

Bearing in mind Eqs. (55) and (56), the eigenvalue problem (54) gives

57 $$\begin{aligned}&\dfrac{1}{f}\left( 3 \tanh (\eta ) \partial _\eta + \partial ^2_\eta \right) f -\dfrac{\tanh ^2 \eta }{g} \left( \dfrac{2}{\tanh \chi }\partial _\chi +\partial ^2_\chi \right) g\nonumber \\&\quad +\dfrac{l(l+1)\tanh ^2 \eta }{ \sinh ^2 \chi } = \lambda R^2, \end{aligned}$$

where we have divided both sides by $\tilde{\phi }(\eta ,\chi )Y^m_l(\theta ,\varphi )$. After having performed some manipulations, the above equation can be solved through the method of separation of variables, yielding the following two ordinary differential equations:

58a $$\begin{aligned} \left( \partial ^2_\eta + 3 \tanh (\eta ) \partial _\eta -\beta \tanh ^2 \eta -\lambda R^2 \right) f(\eta )&=0, \end{aligned}$$

58b $$\begin{aligned} \left( \partial ^2_\chi +\dfrac{2}{\tanh \chi } \partial _\chi -\dfrac{l(l+1)}{\sinh ^2 \chi } - \beta \right) g(\chi )&=0, \end{aligned}$$

$\beta$ being a real-valued constant.

The solution of Eq. (58) and the eigenfunctions of the d’Alembertian operator (53) will be provided in the next sections.

#### The time-like equation

It is instructive to work out the details of the solution of Eq. (58a). If we introduce the variable

59 $$\begin{aligned} w=\tanh \eta \in (-1,1), \end{aligned}$$

then the derivative operators read as

60 $$\begin{aligned} \partial _\eta&= (1-w^2)\partial _w, \nonumber \\ \partial ^2_\eta&= -2w(1-w^2)\partial _w + (1-w^2)^2 \partial ^2_w, \end{aligned}$$

and hence Eq. (58a) becomes

61 $$\begin{aligned} \left[ (1-w^2) \partial ^2_w + w \partial _w - \beta \dfrac{w^2}{1-w^2} -\dfrac{\lambda R^2}{1-w^2} \right] f(w)=0. \end{aligned}$$

If we write

62 $$\begin{aligned} f(w)=(1-w^2)^{3/4}h(w), \end{aligned}$$

then we end up with the general Legendre equation [53, 54]

63 $$\begin{aligned}&(1-w^2)\partial ^2_w h -2w\partial _w h + \left[ \left( \frac{3}{4}+\beta \right) \right. \nonumber \\&\quad \left. -\left( \frac{\frac{9}{4} +\beta +\lambda R^2}{1-w^2}\right) \right] h=0, \end{aligned}$$

which can be solved via the associated Legendre functions of the first and second kind $\mathsf {P}^\mu _\nu (w)$ and $\mathsf {Q}^\mu _\nu (w)$, respectively, having degree $\nu$ and order $\mu$ given by

64a $$\begin{aligned} \nu&= \dfrac{1}{2}\left( 2\sqrt{1+\beta }-1\right) , \end{aligned}$$

64b $$\begin{aligned} \mu&= \dfrac{1}{2}\sqrt{9+4\beta +4\lambda R^2}. \end{aligned}$$

Therefore, bearing in mind Eqs. (59) and (62), the solution of Eq. (58a) in terms of the variable $\eta$ is

65 $$\begin{aligned} f(\eta )= (1-\tanh ^2 \eta )^{3/4}\left[ c_1 \mathsf {P}^\mu _\nu (\tanh \eta ) +c_2 \mathsf {Q}^\mu _\nu (\tanh \eta )\right] , \end{aligned}$$

$c_1$ and $c_2$ being integration constants. Here $\mathsf {Q}^{\mu }_{\nu }$ can equivalently be replaced by $\mathsf {P}^{- \mu }_{\nu }$, which will be done in the following.

#### The radial equation

The solution of (58b) can be written in terms of the Gaussian or ordinary hypergeometric function [53] as

66 $$\begin{aligned} g(\chi )&= \dfrac{\left( 1-\tanh ^2 \chi \right) ^{(1/2)(1-a+b+c)}}{\left( \tanh \chi \right) ^{3/2}} \nonumber \\&\quad \times \Biggl [ c_3 \left( \tanh ^2 \chi \right) ^{a/2} {}_{2}\mathrm {F}_{1}\left( b,c;a;\tanh ^2 \chi \right) \nonumber \\&\quad + c_4 \left( -1\right) ^{1-a}\left( \tanh ^2 \chi \right) ^{1-a/2} \nonumber \\&\quad \times {}_{2}\mathrm {F}_{1} \left( 1-a+b,1-a+c;2-a;\tanh ^2 \chi \right) \Biggr ], \end{aligned}$$

where $c_3$ and $c_4$ are integration constants and

67 $$\begin{aligned} a&\equiv \dfrac{1}{2} \left( 3+2l\right) , \nonumber \\ b&\equiv \dfrac{1}{2} \left( 1+l+\sqrt{1+\beta }\right) , \nonumber \\ c&\equiv \dfrac{1}{2} \left( 2+l+\sqrt{1+\beta }\right) = b + \dfrac{1}{2}. \end{aligned}$$

It is possible to write Eqs. (58b) and (66) in a more convenient form. Indeed, if we introduce the variable (recall that we are considering $\chi >0$)

68 $$\begin{aligned} y=\coth \chi \in (1,+\infty ), \end{aligned}$$

then we have

69 $$\begin{aligned} \partial _\chi&= (1-y^2)\partial _y, \nonumber \\ \partial ^2_\chi&= (1-y^2)^2\partial ^2_y -2y(1-y^2)\partial _y, \end{aligned}$$

and hence Eq. (58b) becomes

70 $$\begin{aligned} (1-y^2)\partial ^2_y g(y)+ \left[ l(l+1)-\dfrac{\beta }{1-y^2}\right] g(y)=0. \end{aligned}$$

The solution of the above equation reads as

71 $$\begin{aligned} g(\chi )=\sqrt{\coth ^2 \chi -1} \left[ c_3 \mathcal {P}^{\tilde{\mu }}_l(\coth \chi ) +c_4 \mathcal {Q}^{\tilde{\mu }}_l(\coth \chi ) \right] , \end{aligned}$$

where we have exploited Eq. (68) and

72 $$\begin{aligned} \tilde{\mu } \equiv \sqrt{1+\beta }. \end{aligned}$$

The physically meaningful solutions can be identified by considering the boundary conditions. Recall that $\chi \in [0,\infty )$ plays the role of a radial variable. In the limit $\chi \rightarrow 0$, we have (B.8)

73 $$\begin{aligned} \mathcal {Q}^{{\tilde{\mu }}}_l(\coth \chi ) \sim (\coth \chi )^{-l-1} \sim \chi ^{l+1} \end{aligned}$$

since $\coth \chi \sim \chi ^{-1}$, while $\mathcal {P}^{\tilde{\mu }}_l(\coth \chi )$ is divergent for $l\geqslant 0$, and finite for $l=0$. Therefore to have non-singular solutions, we should choose the $\mathcal {Q}^{{\tilde{\mu }}}_l$ solutions and reject the $\mathcal {P}^{{\tilde{\mu }}}_l$.

#### The eigenmodes on the spacetime

Thanks to the solutions (65) and (71), the eigenfunctions (55) of the d’Alembertian operator (53) are known. Moreover, Eq. (64b) permits obtaining the explicit expression of the eigenvalue $\lambda$, which reads as

74 $$\begin{aligned} \lambda = \dfrac{ \mu ^2-\beta -\frac{9}{4}}{R^2}. \end{aligned}$$

In order to have oscillatory (square-integrable) solutions, we should assume that the order (64b) of the solution (65) is purely imaginary. Therefore, we set

75 $$\begin{aligned} \mu = \pm is, \end{aligned}$$

where

76 $$\begin{aligned} s= \vert \mu \vert =\sqrt{-\left( \dfrac{9}{4}+\beta +\lambda R^2\right) } \ >0. \end{aligned}$$

Similarly, the order (72) of the solution (71) should be purely imaginary, i.e.

77 $$\begin{aligned} \tilde{\mu }= i q . \end{aligned}$$

for $q\in {\mathbb R}$. This leads to

78 $$\begin{aligned} q^2 = -\left( 1+\beta \right) >0 , \end{aligned}$$

which implies that

79 $$\begin{aligned} \beta < -1. \end{aligned}$$

From Eqs. (76) and (78), the eigenvalue (74) can be written equivalently as

80 $$\begin{aligned} \lambda = \dfrac{ q^2-s^2 -\frac{5}{4}}{R^2}. \end{aligned}$$

As a consequence of Eq. (79), we see that the degree (64a) of the solution (65) is complex and reads as

81 $$\begin{aligned} \nu = -\dfrac{1}{2} \pm iq . \end{aligned}$$

In order not to overcount equivalent solutions, we observe that the $\mathsf {P}^\mu _\nu (\tanh \eta )$ coincide for the two choices of $\nu = -\dfrac{1}{2} \pm iq$. We can therefore make the convention that

82 $$\begin{aligned} \nu = -\dfrac{1}{2} + i|q|. \end{aligned}$$

Collecting Eqs. (75)–(78) jointly with Eqs. (65) and (71), the eigenmodes (55) of the d’Alembertian operator (53) having the appropriate boundary conditions are83recalling that $\chi >0$. The above-defined eigenmodes are regular functions in the limit $\chi \rightarrow 0$. Indeed, due to the relation (B.8), we have

84 $$\begin{aligned} \Upsilon ^{s_\pm ,q}_{l,m}(\chi ) \sim \chi ^{l}, \quad \text{ as } \ \chi \rightarrow 0 . \end{aligned}$$

Now consider the limit $\chi \rightarrow +\infty$, which means that $\coth \chi \rightarrow 1^+$. The functions $\mathcal {Q}^{iq}_l(x)$ are oscillatory but bounded when $x\rightarrow 1^+$ as it can be inferred from Eq. (B.9). In this way, we obtain

85 $$\begin{aligned} \Upsilon ^{s_\pm ,q}_{l,m}\left( \chi \right) \sim \frac{e^{i\chi q}}{\sinh (\chi )}, \quad \text{ as } \ \chi \rightarrow +\infty . \end{aligned}$$

Therefore, the eigenfunctions $\Upsilon ^{s_\pm ,q}_{l,m}\left( \eta ,\chi ,\theta ,\varphi \right)$ are square-integrable oscillatory functions on $\mathcal{M}^{3,1}$, as it should be. Moreover, they are continuous across the BB i.e. at $\eta = 0$.

#### Flat regime

Now consider the following “flat” regime^3^

86 $$\begin{aligned} \text{ FR }: \chi < 1, \quad q \gg l, \end{aligned}$$

where “FR” stands for “flat regime”, and *q* will be a typical momentum. Then the $\mathcal {Q}^{iq}_l$ reduces to the spherical Bessel functions,87This should be expected, since the eigenfunctions (83) should reduce to the standard ones on ${\mathbb R}^{3,1}$. For $\chi \rightarrow 0$, the relation (87) is guaranteed by the standard expansion formulas for $\mathcal {Q}^{iq}_l(\coth \chi )$ and $j_l(q\chi )$;^4^ here, we have verified that it works very well in the range $\chi \in (0,1)$ for $q\gg l$. Moreover, it also holds that

88 $$\begin{aligned} \mathrm{Im}\left[ \frac{\mathcal {Q}^{iq}_l(\coth \chi )}{\sinh \chi } \frac{q^l}{e^{-\pi q} \Gamma (i q + l + 1)} \right] \overset{\chi <1}{\sim } 0, \end{aligned}$$

and hence we can conclude that89Due to the relation [55]

90 $$\begin{aligned} \mathsf {P}^\mu _\nu (x)&\overset{x \rightarrow 1^{-}}{\sim } \dfrac{1}{\Gamma (1-\mu )} \left( \dfrac{1+x}{1-x}\right) ^{\mu /2}, \end{aligned}$$

we easily obtain

91 $$\begin{aligned} \mathsf {P}^{\pm is}_\nu \left( \tanh \eta \right)&\overset{\eta \rightarrow +\infty }{\sim } \dfrac{1}{\Gamma (1 \mp is)} e^{\pm i \eta s}. \end{aligned}$$

Thus, bearing in mind Eqs. (89) and (91), the eigenmodes (83) for large times (i.e., $\eta \rightarrow + \infty$) and in the flat regime (86) become92

### Orthogonality relations

The orthogonality relations for the eigenfunctions (83) of the d’Alembertian operator (53) are written via the *SO*(4, 1)-invariant inner product as

93 $$\begin{aligned}&\langle \Upsilon ^{s^\prime _{+},q^\prime }_{l^\prime ,m^\prime }, \Upsilon ^{s_{+},q}_{l,m}\rangle \left( \eta ,\chi ,\theta ,\varphi \right) := \int \Omega \left[ \Upsilon ^{s^\prime _{+},q^\prime }_{l^\prime ,m^\prime }\left( \eta ,\chi ,\theta ,\varphi \right) \right] ^*\nonumber \\&\quad \times \Bigl [\Upsilon ^{s_{+},q}_{l,m} \left( \eta ,\chi ,\theta ,\varphi \right) \Bigr ], \end{aligned}$$

the symplectic volume form $\Omega$ being given in Eq. (43). Explicitly, we obtain

94 $$\begin{aligned}&\langle \Upsilon ^{s^\prime _{+},q^\prime }_{l^\prime ,m^\prime }, \Upsilon ^{s_{+},q}_{l,m}\rangle = \int _0^{2 \pi }d \varphi \int _0^\pi d \theta \sin \theta \left[ Y^{m^\prime }_{l^\prime }\left( \theta ,\varphi \right) \right] ^*\nonumber \\&\qquad \times \Bigl [Y^{m}_{l}\left( \theta ,\varphi \right) \Bigr ] \nonumber \\&\qquad \times \int ^{+\infty }_{-\infty } d\eta \left[ \mathsf {P}^{ is^\prime }_{\nu ^\prime }\left( \tanh \eta \right) \right] ^* \Bigl [ \mathsf {P}^{ is}_\nu \left( \tanh \eta \right) \Bigr ] \int ^{+\infty }_{0} d \chi \nonumber \\&\qquad \times \left[ \mathcal {Q}^{iq^\prime }_{l^\prime }\left( \coth \chi \right) \right] ^* \Bigl [\mathcal {Q}^{ iq}_l\left( \coth \chi \right) \Bigr ] \nonumber \\&\quad = \delta _{l l^\prime } \delta _{m m^\prime } \int ^{+\infty }_{-\infty } d\eta \left[ \mathsf {P}^{ is^\prime }_{\nu ^\prime }\left( \tanh \eta \right) \right] ^* \Bigl [ \mathsf {P}^{ is}_\nu \left( \tanh \eta \right) \Bigr ] \nonumber \\&\qquad \times \int ^{+\infty }_{0} d \chi \left[ \mathcal {Q}^{iq^\prime }_{l}\left( \coth \chi \right) \right] ^* \Bigl [\mathcal {Q}^{ iq}_l\left( \coth \chi \right) \Bigr ], \end{aligned}$$

where we have exploited the orthogonality of the spherical harmonics [52] and the ensuing Kronecker delta factor $\delta _{l l^\prime }$. We first consider the integral involving the $\chi$ variable. If we define $\xi = \tanh \chi$, then we obtain

95 $$\begin{aligned}&\int ^{+\infty }_{0} d \chi \left[ \mathcal {Q}^{iq^\prime }_{l}\left( \coth \chi \right) \right] ^* \Bigl [\mathcal {Q}^{ iq}_l\left( \coth \chi \right) \Bigr ] \nonumber \\&\quad =\int _0^1 \dfrac{d \xi }{1-\xi ^2}\left[ \mathcal {Q}^{iq^\prime }_{l} (1/\xi )\right] ^* \mathcal {Q}^{ iq}_l(1/\xi ), \end{aligned}$$

which, via the substitution $x = 1/\xi$, yields

96 $$\begin{aligned}&\int _0^1 \dfrac{d \xi }{1-\xi ^2}\left[ \mathcal {Q}^{iq^\prime }_{l} (1/\xi )\right] ^* \mathcal {Q}^{ iq}_l(1/\xi ) \nonumber \\&\quad =-\int _1^{+\infty } \dfrac{dx}{1-x^2} \left[ \mathcal {Q}^{iq^\prime }_{l} (x)\right] ^* \mathcal {Q}^{ iq}_l(x). \end{aligned}$$

The above integral can be worked out with the help of the results of Appendix B, where we have shown that

97 $$\begin{aligned}&\int _1^{+\infty } \dfrac{dx}{1-x^2} \mathcal {Q}^{iq^\prime }_{l} (x) \mathcal {Q}^{ iq}_l(x)\nonumber \\&\quad =-\dfrac{\left( \pi /2\right) ^2}{q \sinh \left( \pi q\right) } \delta (q+q^\prime ), \quad (l>-3). \end{aligned}$$

The above relation leads to

98 $$\begin{aligned} -\int _1^{+\infty } \dfrac{dx}{1-x^2} \mathcal {Q}^{-iq^\prime }_{l} (x) \mathcal {Q}^{ iq}_l(x)=\dfrac{\left( \pi /2\right) ^2}{q \sinh \left( \pi q\right) } \delta (q-q^\prime ), \end{aligned}$$

which in turn means that, for any real $q,q^\prime$,

99 $$\begin{aligned}&\int ^{+\infty }_{0} d \chi \left[ \mathcal {Q}^{iq^\prime }_{l}\left( \coth \chi \right) \right] ^* \Bigl [\mathcal {Q}^{ iq}_l\left( \coth \chi \right) \Bigr ]\nonumber \\&\quad = \dfrac{\left( \pi /2\right) ^2 e^{-2 \pi q}}{q \sinh \left( \pi q\right) } \delta (q-q^\prime ), \end{aligned}$$

where we have exploited (see Eqs. (B.24)–(B.26))

100 $$\begin{aligned} \left[ \mathcal {Q}^{iq^\prime }_l(x)\right] ^* = e^{-2 \pi q^\prime } \mathcal {Q}^{-iq^\prime }_{l}(x). \end{aligned}$$

By virtue of the identity (99), Eq. (94) gives

101 $$\begin{aligned}&\langle \Upsilon ^{s^\prime _{+},q^\prime }_{l^\prime ,m^\prime }, \Upsilon ^{s_{+},q}_{l,m}\rangle \nonumber \\&\quad = \dfrac{e^{-2 \pi q}\left( \pi /2\right) ^2}{q \sinh \left( \pi q\right) } \delta _{l l^\prime } \delta _{m m^\prime } \delta (q-q^\prime )\nonumber \\&\qquad \times \int ^{+\infty }_{-\infty } d\eta \left[ \mathsf {P}^{ is^\prime }_{\nu ^\prime }\left( \tanh \eta \right) \right] ^* \Bigl [ \mathsf {P}^{ is}_\nu \left( \tanh \eta \right) \Bigr ] \nonumber \\&\quad =\dfrac{e^{-2 \pi q}\left( \pi /2\right) ^2}{q \sinh \left( \pi q\right) } \delta _{l l^\prime } \delta _{m m^\prime } \delta (q-q^\prime ) \nonumber \\&\qquad \times \int ^{+\infty }_{-\infty } d\eta \left[ \mathsf {P}^{ is^\prime }_{\nu }\left( \tanh \eta \right) \right] ^* \Bigl [ \mathsf {P}^{ is}_\nu \left( \tanh \eta \right) \Bigr ]. \end{aligned}$$

We recall that the expression of the associated Legendre function of the first kind with a generic degree $\nu$ and argument *x* lying in the interval $(-1,1)$ is [53, 55]

102 $$\begin{aligned} \mathsf {P}^{ is^\prime }_\nu \left( x \right)&= \dfrac{1}{\Gamma (1-is^\prime )}\left( \frac{1+x}{1-x}\right) ^{is^\prime /2}\nonumber \\&\quad \times {}_{2}\mathrm {F}_{1}\left( \nu +1,-\nu ;1-is^\prime ;\tfrac{1}{2}-\tfrac{1}{2}x\right) ,\nonumber \\&\quad (-1<x<1), \end{aligned}$$

${}_{2}\mathrm {F}_{1}\left( a,b;c;x\right)$ being, like before, the Gaussian (or ordinary) hypergeometric function and $\Gamma (x)$ the gamma function. In our case, the degree $\nu$ of $\mathsf {P}^{ is^\prime }_{\nu }\left( \tanh \eta \right)$ assumes the form given in Eq. (82). For this choice of $\nu$, we find

103 $$\begin{aligned} \left[ \mathsf {P}^{ is^\prime }_{\nu }\left( \tanh \eta \right) \right] ^* = \mathsf {P}^{- is^\prime }_{\nu }\left( \tanh \eta \right) , \end{aligned}$$

where we have exploited Eq. (102) jointly with the property ${}_{2}\mathrm {F}_{1}\left( a,b;c;x\right) ={}_{2}\mathrm {F}_{1}\left( b,a;c;x\right)$. With this result in mind, it follows from Eq. (101) that

104 $$\begin{aligned} \langle \Upsilon ^{s^\prime _{+},q^\prime }_{l^\prime ,m^\prime }, \Upsilon ^{s_{+},q}_{l,m}\rangle&= \dfrac{e^{-2 \pi q}\left( \pi /2\right) ^2}{q \sinh \left( \pi q\right) } \delta _{l l^\prime } \delta _{m m^\prime } \delta (q-q^\prime )\nonumber \\&\quad \times \int ^{+\infty }_{-\infty } d\eta \, \mathsf {P}^{-is^\prime }_{\nu }\left( \tanh \eta \right) \mathsf {P}^{ is}_\nu \left( \tanh \eta \right) \nonumber \\&=\dfrac{e^{-2 \pi q}\left( \pi /2\right) ^2}{q \sinh \left( \pi q\right) } \delta _{l l^\prime } \delta _{m m^\prime } \delta (q-q^\prime ) \nonumber \\&\quad \times \int ^{1}_{-1} \dfrac{dy}{1-y^2} \mathsf {P}^{-is^\prime }_{\nu }\left( y \right) \mathsf {P}^{ is}_\nu \left( y \right) , \end{aligned}$$

where we have employed the substitution $y=\tanh \eta$. This last integral can be calculated with the help of following result:

105 $$\begin{aligned}&\int ^1_{-1} \dfrac{dy}{1-y^2}\mathsf {P}^{is^\prime }_\nu (y) \mathsf {P}^{is}_\nu (y) \nonumber \\&\quad = -\dfrac{2 \pi \sin (\pi \nu )}{s \sinh (\pi s)}\dfrac{1}{\Gamma (1+\nu -is)\Gamma (-\nu -is)}\delta (s-s^\prime ) \nonumber \\&\qquad +2\left[ \dfrac{\sinh (\pi s)}{s}+\dfrac{\sin ^2(\pi \nu )}{s \sinh (\pi s)}\right] \delta (s+s^\prime ), \end{aligned}$$

which has been proved in Ref. [56] (see Appendix B for further details). Therefore, bearing in mind the above formula along with Eq. (82), from Eq. (104) we finally obtain the sought-after orthogonality relations

106 $$\begin{aligned} \langle \Upsilon ^{s^\prime _{+},q^\prime }_{l^\prime ,m^\prime }, \Upsilon ^{s_{+},q}_{l,m}\rangle&= \int \Omega \left[ \Upsilon ^{s^\prime _{+},q^\prime }_{l^\prime ,m^\prime }\right] ^* \Bigl [\Upsilon ^{s_{+},q}_{l,m} \Bigr ] \nonumber \\&=\dfrac{e^{-2 \pi q}\left( \pi /2\right) ^2}{q \sinh \left( \pi q\right) } \delta _{l l^\prime } \delta _{m m^\prime } \delta (q-q^\prime ) \nonumber \\&\quad \times \Biggl [a(q,s)\delta (s+s^\prime ) + b(q,s)\delta (s-s^\prime )\Biggr ], \end{aligned}$$

where

107a $$\begin{aligned}&a(q,s) \nonumber \\&\quad = \dfrac{2 \pi \cosh (\pi q)}{s \sinh (\pi s)}\dfrac{1}{\Gamma \left( iq-is+1/2\right) \Gamma (-iq-is+1/2)}\nonumber \\&\quad =a(q,-s)^*, \end{aligned}$$

107b $$\begin{aligned}&b(q,s) = \dfrac{2 \sinh (\pi s)}{s}\left[ 1+\dfrac{\cosh ^2(\pi q)}{\sinh ^2(\pi s)}\right] =b(q,-s). \end{aligned}$$

### Completeness relation for the $\mathcal {Q}^{iq}_{\nu }$

As a consequence of (99), we obtain the following completeness relation for the $\mathcal {Q}^{iq}_{\nu }(\coth \chi )$ in the interval $(1,+\infty )$:

108 $$\begin{aligned}&\int _{-\infty }^{+\infty } dq\, \dfrac{q \sinh \left( \pi q\right) }{e^{-2 \pi q}\left( \pi /2\right) ^2}\, \mathcal {Q}^{iq}_{l}(\coth \chi ^\prime ) \left[ \mathcal {Q}^{iq}_{l}(\coth \chi )\right] ^* \nonumber \\&\quad = \delta (\chi -\chi ^\prime ) . \end{aligned}$$

To prove this, it suffices to multiply this equation with $\mathcal {Q}^{iq^\prime }_{l}(\coth \chi )$ and integrate over $\int _0^{+\infty } d\chi$ using (99). This completeness relation allows to represent any (square-integrable) function $\phi (\chi )$ defined in the interval $(0,+\infty )$ as superposition of $\mathcal {Q}^{iq}_{l}$ modes,

109 $$\begin{aligned} \phi (\chi ) = \int _{-\infty }^{+\infty } dq\, \dfrac{q \sinh \left( \pi q\right) }{e^{-2 \pi q}\left( \pi /2\right) ^2}\, {\hat{\phi }}(q)\, \mathcal {Q}^{iq}_{l}(\coth \chi ), \end{aligned}$$

where ${\hat{\phi }}(q)$ is given by

110 $$\begin{aligned} {\hat{\phi }}(q) = \int _0^{+\infty } d\chi \, \phi (\chi ) \left[ \mathcal {Q}^{iq}_{l}(\coth \chi )\right] ^*. \end{aligned}$$

The factor $\dfrac{q \sinh \left( \pi q\right) }{e^{-2 \pi q}\left( \pi /2\right) ^2}$ can of course be absorbed via a suitable redefinition of ${\hat{\phi }}(q)$.

### Quantization and matrix version

The above discussion regarding the eigenmodes of the d’Alembertian (53) is completely classical, even though we are claiming to work in the framework of matrix models. This may seem suspect, but it can be fully justified as follows. Since the underlying fuzzy hyperboloid $H^4_n$ is a quantized coadjoint orbit, there is an *SO*(4, 1)-equivariant quantization map^5^

111 $$\begin{aligned} \mathcal{Q}: \mathcal{C}(H^4) \rightarrow \mathrm {End}(\mathcal{H}) \end{aligned}$$

which establishes an isometric equivalence between commutative and the fuzzy “functions”, and maps the matrix d’Alembertian $\Box$ to the above semi-classical version. Therefore all the eigenmodes and eigenvalues computed in the classical case carry over without corrections to the fuzzy case, *in the free theory*. This justifies the classical computations in this work.

## Path integral quantization

In this section we compute the propagator of a scalar field $\phi (x)$ in the FLRW geometry (8). After having investigated the action in Sect. 5.1, the propagator of $\phi (x)$ is explicitly worked out in Sect. 5.2.

### The action

In the semi-classical limit, the action for a scalar field $\phi (x)$ having mass *m* can be written as

112 $$\begin{aligned} S_{\varepsilon }\left[ \phi \right] = \int \Omega \phi ^*(x)\left( -\Box -m^2 + i \varepsilon \right) \phi (x), \end{aligned}$$

where the expression of $\Omega$ can read from Eq. (43) and, as usual, $\varepsilon$ is a small positive number which should be let tend to zero after integration. The exact matrix version of the action has the same form with the trace $\mathrm {Tr}$ replacing the integral $\int d\Omega$, and the $i\varepsilon$ term ensures that the matrix path integral $\int D\phi \, e^{i S}$ is well-defined [57]. We can evaluate $S_{\varepsilon }\left[ \phi \right]$ by employing the following decomposition of $\phi (x)$ in the basis of eigenmodes (83):

113 $$\begin{aligned} \phi (x)= \sum _{l,m}\int ds dq \Bigl [\phi ^+_{s,q,l,m} \Upsilon ^{s_{+},q}_{l,m}(x)+\phi ^-_{s,q,l,m} \Upsilon ^{s_{-},q}_{l,m}(x) \Bigr ], \end{aligned}$$

$\phi ^+_{s,q,l,m},\phi ^-_{s,q,l,m}$ being the coefficients of such a decomposition. In order to ease the notation, in our forthcoming calculations we will set

114 $$\begin{aligned} \phi ^{\pm }&\equiv \phi ^{\pm }_{s,q,l,m} , \nonumber \\ \phi ^{\prime \pm }&\equiv \phi ^{\pm }_{s^\prime ,q^\prime ,l^\prime ,m^\prime } . \end{aligned}$$

Bearing in mind Eqs. (80) and (113), the action (112) becomes

115 $$\begin{aligned}&S_{\varepsilon }\left[ \phi \right] = \sum _{l,m}\int ds dq \left[ -\dfrac{1}{R^2}\left( q^2-s^2-\dfrac{5}{4}\right) -m^2 + i \varepsilon \right] \nonumber \\&\qquad \times \int \Omega \phi ^*(x) \left[ \left( \phi ^+\right) \Upsilon ^{s_{+},q}_{l,m}(x)+\left( \phi ^-\right) \Upsilon ^{s_{-},q}_{l,m}(x) \right] \nonumber \\&\quad = \sum _{l,m}\sum _{l^\prime ,m^\prime }\int ds dq ds^\prime dq^\prime \nonumber \\&\qquad \times \left[ -\dfrac{1}{R^2}\left( q^2-s^2-\dfrac{5}{4}\right) -m^2 + i \varepsilon \right] \nonumber \\&\qquad \times \int \Omega \left[ \left( \phi ^{\prime +}\right) \Upsilon ^{s^\prime _{+},q^\prime }_{l^\prime ,m^\prime }(x)+\left( \phi ^{\prime -}\right) \Upsilon ^{s^\prime _{-},q^\prime }_{l^\prime ,m^\prime }(x) \right] ^* \nonumber \\&\qquad \times \left[ \left( \phi ^+\right) \Upsilon ^{s_{+},q}_{l,m}(x)+\left( \phi ^- \right) \Upsilon ^{s_{-},q}_{l,m}(x) \right] . \end{aligned}$$

Thanks to the orthogonality relations (106), the above equation gives

116 $$\begin{aligned}&S_{\varepsilon }\left[ \phi \right] \nonumber \\&= \sum _{l,m}\sum _{l^\prime ,m^\prime }\int ds dq ds^\prime dq^\prime \left[ -\dfrac{1}{R^2}\left( q^2-s^2-\dfrac{5}{4}\right) \right. \nonumber \\&\quad \left. -m^2 + i \varepsilon \right] \Biggl [ \left( \phi ^{\prime +}\right) ^* \phi ^+ \langle \Upsilon ^{s^\prime _{+},q^\prime }_{l^\prime ,m^\prime }, \Upsilon ^{s_{+},q}_{l,m} \rangle \nonumber \\&\quad + \left( \phi ^{\prime +}\right) ^* \phi ^- \langle \Upsilon ^{s^\prime _{+},q^\prime }_{l^\prime ,m^\prime }, \Upsilon ^{s_{-},q}_{l,m} \rangle + \left( \phi ^{\prime -}\right) ^* \phi ^+ \langle \Upsilon ^{s^\prime _{-},q^\prime }_{l^\prime ,m^\prime }, \Upsilon ^{s_{+},q}_{l,m} \rangle \nonumber \\&\quad +\left( \phi ^{\prime -}\right) ^* \phi ^- \langle \Upsilon ^{s^\prime _{-},q^\prime }_{l^\prime ,m^\prime }, \Upsilon ^{s_{-},q}_{l,m} \rangle \Biggr ] \nonumber \\&= \sum _{l,m}\sum _{l^\prime ,m^\prime }\int ds dq ds^\prime dq^\prime \Biggl \{ \left[ -\dfrac{1}{R^2}\left( q^2-s^2-\dfrac{5}{4}\right) \right. \nonumber \\&\quad \left. -m^2 + i \varepsilon \right] \dfrac{e^{-2\pi q}(\pi /2)^2 \, \delta _{l l^\prime } \delta _{m m^\prime }}{q \sinh (\pi q)} \delta (q-q^\prime ) \nonumber \\&\quad \times \delta (s-s^\prime ) \Bigl [\left( \phi ^{\prime +}\right) ^* \left( \phi ^+\right) b(q,s)+\left( \phi ^{\prime +}\right) ^* \left( \phi ^-\right) a(q,-s)\nonumber \\&\quad +\left( \phi ^{\prime -}\right) ^* \left( \phi ^+\right) a(q,s) +\left( \phi ^{\prime -}\right) ^* \left( \phi ^-\right) b(q,-s)\Bigr ]\Biggr \}, \end{aligned}$$

where in the last passage we have exploited the fact that all terms proportional to $\delta (s+s^\prime )$ give a vanishing contribution, since both *s* and $s^\prime$ are positive (cf. Eq. (76)). If we introduce the square matrix

117 $$\begin{aligned} \mathscr {B}(q,s)= \begin{bmatrix} b(q,s) &{} a(q,-s)\\ a(q,s) &{} b(q,-s) \end{bmatrix}, \end{aligned}$$

then we can write

118 $$\begin{aligned}&\begin{bmatrix} \left( \phi ^{\prime +}\right) ^* \left( \phi ^{\prime -}\right) ^* \end{bmatrix} \mathscr {B}(q,s) \begin{bmatrix} \phi ^+ \\ \phi ^- \end{bmatrix} =\left( \phi ^{\prime +}\right) ^* \left( \phi ^+\right) b(q,s)\nonumber \\&\quad +\left( \phi ^{\prime +}\right) ^* \left( \phi ^-\right) a(q,-s) \nonumber \\&\quad +\left( \phi ^{\prime -}\right) ^* \left( \phi ^+\right) a(q,s) +\left( \phi ^{\prime -}\right) ^* \left( \phi ^-\right) b(q,-s), \end{aligned}$$

and hence, in conclusion, the action assumes the form

119 $$\begin{aligned}&S_{\varepsilon }\left[ \phi \right] = \sum _{l,m}\sum _{l^\prime ,m^\prime }\int ds dq ds^\prime dq^\prime \left[ -\dfrac{1}{R^2}\left( q^2-s^2-\dfrac{5}{4}\right) \right. \nonumber \\&\quad \left. -m^2 + i \varepsilon \right] \dfrac{e^{-2\pi q}(\pi /2)^2 }{q \sinh (\pi q)} \nonumber \\&\quad \times \delta _{l l^\prime } \delta _{m m^\prime } \delta (q-q^\prime ) \delta (s-s^\prime ) \begin{bmatrix} \left( \phi ^{\prime +}\right) ^*&\left( \phi ^{\prime -}\right) ^* \end{bmatrix}\nonumber \\&\quad \times \mathscr {B}(q,s) \begin{bmatrix} \phi ^+ \\ \phi ^- \end{bmatrix}. \end{aligned}$$

### The propagator in momentum space and position space

In the following, we evaluate the propagator of the massive scalar field $\phi (x)$. We first provide the general expressions for the propagator both in momentum space and position space. The late-time propagator is then elaborated in Sect. 5.2.1, recalling also a related topic – i.e. the expansion of plane waves in terms of spherical harmonics – in Sect. 5.2.2. Finally, in Sect. 5.2.3 we compute the propagator across the BB.

Starting from the action (119) and adopting the compact notation (cf. Eq. (114))

120 $$\begin{aligned} \Phi ^{\pm }&\equiv \begin{bmatrix} \phi ^+ \\ \phi ^- \end{bmatrix}, \nonumber \\ \left( \Phi ^{\prime \pm }\right) ^{\dagger }&\equiv \begin{bmatrix} \left( \phi ^{\prime +}\right) ^*&\left( \phi ^{\prime -}\right) ^* \end{bmatrix}, \end{aligned}$$

the propagator in momentum space reads as

121 $$\begin{aligned} \left\langle \left( \Phi ^{\pm }\right) \left( \Phi ^{\prime \pm }\right) ^{\dagger } \right\rangle&= \delta _{l l^\prime } \delta _{m m^\prime } \delta \left( q-q^\prime \right) \delta \left( s-s^\prime \right) \nonumber \\&\times \dfrac{1}{\dfrac{1}{R^2}\left( s^2 - q^2 +\dfrac{5}{4}\right) -m^2 + i \varepsilon } \nonumber \\&\times \dfrac{4q \sinh (\pi q)}{e^{-2 \pi q}\pi ^2} \left[ \mathscr {B}(q,s)\right] ^{-1}, \end{aligned}$$

where

122 $$\begin{aligned} \left[ \mathscr {B}(q,s)\right] ^{-1} =\dfrac{1}{\det \left[ \mathscr {B}(q,s)\right] } \begin{bmatrix} b(q,-s) &{} -a(q,-s) \\ -a(q,s) &{} b(q,s) \end{bmatrix}, \end{aligned}$$

and (see Eq. (107))

123 $$\begin{aligned} \det \left[ \mathscr {B}(q,s)\right] = \dfrac{2}{s^2} \left[ \cosh \left( 2 \pi q\right) +\cosh \left( 2 \pi s\right) \right] . \end{aligned}$$

Therefore, bearing in mind Eq. (121), the local propagator in position space reads as

124 $$\begin{aligned}&\langle \phi (x) \phi ^*(x^\prime )\rangle \nonumber \\&= \sum _{l,m}\sum _{l^\prime ,m^\prime } \int ds dq ds^\prime dq^\prime \begin{bmatrix}&\Upsilon ^{s_+,q}_{l,m} (x) \Upsilon ^{s_-,q}_{l,m}(x) \end{bmatrix}\nonumber \\&\quad \times \left\langle \left( \Phi ^{\pm }\right) \left( \Phi ^{\prime \pm }\right) ^{\dagger } \right\rangle \begin{bmatrix} \left( \Upsilon ^{s^\prime _{+},q^\prime }_{l^\prime ,m^\prime } \left( x^\prime \right) \right) ^* \\ \left( \Upsilon ^{s^\prime _{-},q^\prime }_{l^\prime ,m^\prime } \left( x^\prime \right) \right) ^* \end{bmatrix}. \end{aligned}$$

#### The propagator in the flat regime and with $\eta \rightarrow + \infty$

We are mainly interested in the local propagator for distances far below the curvature scale, but keeping the oscillating nature of the modes in the late-time regime $\eta \rightarrow +\infty$. This is the flat regime FR defined in (86), where the eigenmodes (83) reduce to (92). It then follows from Eq. (124) that the late-time local propagator can be written as the sum of a leading piece and a subleading part, i.e.,125where “L” and “SL” stand for “leading” and “subleading”, respectively. The leading term reads as^6^

126 $$\begin{aligned}&\langle \phi (x) \phi ^*(x^\prime )\rangle ^{\eta \rightarrow + \infty ,\mathrm{FR}}_{\mathrm{L}} = \dfrac{2R^2}{\pi ^3} \sum _{l,m} \dfrac{ Y^m_l(\theta ,\varphi )\left[ Y^m_l(\theta ^\prime ,\varphi ^\prime )\right] ^*}{\sqrt{\left( \cosh ^3 \eta \right) \left( \cosh ^3 \eta ^\prime \right) }} \nonumber \\&\quad \times \int ds dq \dfrac{j_l\left( q \chi \right) j_l\left( q \chi ^\prime \right) }{q^{2l}\left( s^2 - q^2 + \dfrac{5}{4}-m^2 R^2 + i \varepsilon \right) } \nonumber \\&\quad \times 2 \cos \left[ s\left( \eta - \eta ^\prime \right) \right] \left| \Gamma \left( iq+l+1\right) \right| ^2 q \sinh (\pi q). \end{aligned}$$

The above equation can be simplified by means of the identity

127 $$\begin{aligned} \left| \Gamma (iq+l+1) \right| ^2 = \dfrac{\pi }{ q \sinh (\pi q)} \prod _{n=0}^{l} \left[ q^2 + \left( l-n\right) ^2\right] , \end{aligned}$$

which can be proved via the recurrence relation $\Gamma (1+z)=z \Gamma (z)$ [53] along with Eq. (B.2a). Moreover, the formula (127) leads to (cf. (86))

128 $$\begin{aligned} q \sinh (\pi q) \left| \Gamma \left( iq+l+1\right) \right| ^2 \overset{q \gg l}{\sim } \pi \, q^{2l+2}. \end{aligned}$$

The restriction to $q \gg l$ means that we ignore the extreme IR regime of the propagator, which is justified for the typical applications of (quantum) field theory.

Therefore, by exploiting (128) and after some calculation, Eq. (126) becomes

129 $$\begin{aligned}&\langle \phi (x) \phi ^*(x^\prime )\rangle ^{\eta \rightarrow + \infty ,\mathrm{FR}}_{\mathrm{L}} = \dfrac{2R^2}{\pi ^2}\nonumber \\&\qquad \times \sum _{l,m} \dfrac{ Y^m_l(\theta ,\varphi )\left[ Y^m_l(\theta ^\prime ,\varphi ^\prime )\right] ^*}{\sqrt{\left( \cosh ^3 \eta \right) \left( \cosh ^3 \eta ^\prime \right) }} \int ^{+ \infty }_{-\infty } ds\, e^{i s\left( \eta - \eta ^\prime \right) } \nonumber \\&\qquad \times \int ^{+ \infty }_{-\infty } dq \dfrac{q^2 j_l\left( q \chi \right) j_l\left( q \chi ^\prime \right) }{\left( s^2 - q^2 + \dfrac{5}{4} -m^2 R^2 + i \varepsilon \right) } \nonumber \\&\quad =\dfrac{4R^2}{\pi ^2} \sum _{l,m} \dfrac{ Y^m_l(\theta ,\varphi )\left[ Y^m_l(\theta ^\prime ,\varphi ^\prime )\right] ^*}{\sqrt{\left( \cosh ^3 \eta \right) \left( \cosh ^3 \eta ^\prime \right) }} \int ^{+ \infty }_{-\infty } ds\, e^{i s\left( \eta - \eta ^\prime \right) } \nonumber \\&\qquad \times \int ^{+ \infty }_{0} dq \dfrac{q^2 j_l\left( q \chi \right) j_l\left( q \chi ^\prime \right) }{\left( s^2 - q^2 + \dfrac{5}{4}-m^2 R^2 + i \varepsilon \right) }, \end{aligned}$$

where we have taken into account that the integrand is an even function of *q* due to the property $j_l(-x)=(-1)^l j_l(x)$. Up to the $\eta$-dependent normalization factor which reflects the cosmic expansion, we recover precisely the Feynman propagator on a flat 3 + 1-dimensional Minkowski space, including the appropriate $i\varepsilon$ prescription which ensures local causality and determines the arrow of time along growing $\eta$.

The subleading contribution occurring in Eq. (125) is

130 $$\begin{aligned}&\langle \phi (x) \phi ^*(x^\prime )\rangle ^{\eta \rightarrow + \infty ,\mathrm{FR}}_{\mathrm{SL}} = \dfrac{4R^2}{\pi ^5} \sum _{l,m} \dfrac{ Y^m_l(\theta ,\varphi )\left[ Y^m_l(\theta ^\prime ,\varphi ^\prime )\right] ^*}{\sqrt{\left( \cosh ^3 \eta \right) \left( \cosh ^3 \eta ^\prime \right) }} \nonumber \\&\quad \times \int ds dq \dfrac{j_l\left( q \chi \right) j_l\left( q \chi ^\prime \right) s \sinh (\pi s)}{q^{2l}\left( s^2 - q^2 + \dfrac{5}{4} -m^2 R^2 + i \varepsilon \right) } \nonumber \\&\quad \times q \sinh (\pi q) \cosh (\pi q) \left| \Gamma \left( iq+l+1\right) \right| ^2 \nonumber \\&\quad \times \mathrm{Re} \left[ e^{is\left( \eta +\eta ^\prime \right) }\Gamma \left( \dfrac{1}{2}-iq-is\right) \right. \nonumber \\&\quad \times \left. \Gamma \left( \dfrac{1}{2}+iq-is\right) \Gamma ^2\left( is\right) \right] . \end{aligned}$$

By exploiting Eq. (128) and after some arrangement, we find

131 $$\begin{aligned}&\langle \phi (x) \phi ^*(x^\prime )\rangle ^{\eta \rightarrow + \infty ,\mathrm{FR}}_{\mathrm{SL}} = \dfrac{2R^2}{\pi ^4} \nonumber \\&\quad \times \sum _{l,m} \dfrac{ Y^m_l(\theta ,\varphi )\left[ Y^m_l(\theta ^\prime ,\varphi ^\prime )\right] ^*}{\sqrt{\left( \cosh ^3 \eta \right) \left( \cosh ^3 \eta ^\prime \right) }} \int ^{+ \infty }_{-\infty } ds \, e^{is\left( \eta +\eta ^\prime \right) } \nonumber \\&\qquad \times \int ^{+ \infty }_{-\infty }dq \dfrac{j_l\left( q \chi \right) j_l\left( q \chi ^\prime \right) q^2 s \cosh (\pi q) \sinh (\pi s)}{\left( s^2 - q^2 + \dfrac{5}{4} -m^2 R^2 + i \varepsilon \right) } \nonumber \\&\qquad \times \Gamma \left( \dfrac{1}{2}-iq-is\right) \Gamma \left( \dfrac{1}{2}+iq-is\right) \Gamma ^2\left( is\right) \nonumber \\&\quad =\dfrac{4R^2}{\pi ^4} \sum _{l,m} \dfrac{ Y^m_l(\theta ,\varphi )\left[ Y^m_l(\theta ^\prime ,\varphi ^\prime )\right] ^*}{\sqrt{\left( \cosh ^3 \eta \right) \left( \cosh ^3 \eta ^\prime \right) }} \int ^{+ \infty }_{-\infty } ds \, e^{is\left( \eta +\eta ^\prime \right) } \nonumber \\&\qquad \times \int ^{+ \infty }_{0}dq \dfrac{j_l\left( q \chi \right) j_l\left( q \chi ^\prime \right) q^2 s \cosh (\pi q) \sinh (\pi s)}{\left( s^2 - q^2 + \dfrac{5}{4} -m^2 R^2 + i \varepsilon \right) } \nonumber \\&\qquad \times \Gamma \left( \dfrac{1}{2}-iq-is\right) \Gamma \left( \dfrac{1}{2}+iq-is\right) \Gamma ^2\left( is\right) . \end{aligned}$$

The last expression depends on the sum $\eta + \eta ^\prime$ and hence it is rapidly oscillating at late times (i.e., for $\eta , \eta ^\prime \rightarrow +\infty$). This justifies the fact that Eq. (131) gives a subleading contribution with respect to Eq. (129), which, on the contrary, depends on the difference $\eta - \eta ^\prime$. However, a crucial consistency check consists in proving that the leading term (129) has, apart from the normalization factor, the form of the usual local Feynman propagator on a flat four-dimensional spacetime. This task is fulfilled in the Sect. 5.2.2.

#### Plane-wave expansion in terms of the spherical harmonics

The scalar plane waves can be expanded in ${\mathbb R}^3$ in terms of spherical harmonics via the Rayleigh equation [58]

132 $$\begin{aligned} e^{i \vec q \cdot \vec x} = 4\pi \sum \limits _{l,m} i^l j_l(q r) Y_{l}^m({\hat{q}}) \left[ Y_{l}^m({\hat{x}})\right] ^*, \end{aligned}$$

where $q=|\vec q|, \ r=|\vec x|$, ${\hat{x}} = \frac{\vec x}{r}$, ${\hat{q}} = \frac{\vec q}{q}$, and $j_l(q r)$ are the spherical Bessel functions [53]. In the above equation, the complex conjugation can be interchanged between the two spherical harmonics due to the symmetry of the scalar product $\vec q \cdot \vec x$. Therefore, we have

133 $$\begin{aligned} e^{i \vec q \cdot (\vec x - \vec x^\prime )}&= (4\pi )^2\sum \limits _{l,m} \sum \limits _{l^\prime ,m^\prime } \left( i\right) ^{l-l^\prime } j_l(q r) j_{l^\prime }\left( q r^\prime \right) Y_{l}^m({\hat{q}})\nonumber \\&\quad \times \left[ Y_{l^\prime }^{m^\prime }({\hat{q}})\right] ^* \left[ Y_{l}^m({\hat{x}})\right] ^* Y_{l^\prime }^{m^\prime }({\hat{x}}^\prime ), \end{aligned}$$

recalling that $Y_{l}^m(-{\hat{x}}^\prime ) = (-1)^l Y_{l}^m({\hat{x}}^\prime )$. Now we integrate this relation over all $\vec q$ with fixed radius *q*:

134 $$\begin{aligned}&\int \limits _{S_q^2}d\Omega _q e^{i \vec q \cdot (\vec x - \vec x^\prime )} = (4\pi )^2\sum \limits _{l,m} \sum \limits _{l^\prime ,m^\prime } \left( i\right) ^{l-l^\prime } j_l(q r) j_{l^\prime }(q r^\prime ) \nonumber \\&\qquad \times \left[ Y_{l}^m({\hat{x}})\right] ^*Y_{l^\prime }^{m^\prime }({\hat{x}}^\prime ) \int \limits _{S_q^2}d\Omega _q Y_{l}^m({\hat{q}})\left[ Y_{l^\prime }^{m^\prime }({\hat{q}})\right] ^* \nonumber \\&\quad = (4\pi )^2\sum \limits _{l,m} j_l(q r) j_{l}(q r^\prime ) \left[ Y_{l}^m({\hat{x}})\right] ^* Y_{l}^{m}({\hat{x}}^\prime ), \end{aligned}$$

using the orthogonality of the spherical harmonics. Therefore, we can write e.g. the propagator in the form

135 $$\begin{aligned}&\int d^3q\, \frac{e^{i \vec q \cdot (\vec x - \vec x^\prime )}}{s^2 - q^2-M^2} \nonumber \\&\quad = \int _0^{+\infty } dq \ q^2 \int \limits _{S_q^2}d\Omega _q \frac{e^{i \vec q \cdot (\vec x - \vec x^\prime )}}{s^2 - q^2 -M^2} \nonumber \\&\quad = (4\pi )^2 \sum \limits _{l,m} \left[ Y_{l}^m({\hat{x}})\right] ^* Y_{l}^{m}({\hat{x}}^\prime ) \nonumber \\&\qquad \times \int _0^{+\infty } dq \frac{q^2}{s^2 - q^2-M^2} j_l(q r) j_{l}(q r^\prime ). \end{aligned}$$

The analogy with Eq. (129) should be noted.

#### The propagator in the flat regime and with $\eta \rightarrow 0$

The behaviour of the scalar field (cf. Eq. (112)) near the BB can be inferred from the form assumed by its propagator (124) for small times, i.e., when $\eta \rightarrow 0$. Here, we will also employ the flat regime (86).

The definition of the associated Legendre function of the first kind $\mathsf {P}^{\mu }_{\nu }\left( x\right)$ having $x \in (-1,1)$ [53]

136 $$\begin{aligned} \mathsf {P}^{\mu }_{\nu }\left( x\right)&= \dfrac{1}{\Gamma (1-\mu )}\left( \frac{1+x}{1-x}\right) ^{\mu /2}\nonumber \\&\quad \times {}_{2}\mathrm {F}_{1}\left( -\nu ,\nu +1;1-\mu ;\tfrac{1}{2}-\tfrac{1}{2}x\right) , \end{aligned}$$

jointly with Eq. (82), leads to the expression

137 $$\begin{aligned}&\mathsf {P}^{\pm is}_{\nu }\left( \tanh \eta \right) \nonumber \\&\quad =\dfrac{{}_{2}\mathrm {F}_{1}\left( \dfrac{1}{2}-i q ,\dfrac{1}{2}+i q ; 1 \mp is;\dfrac{1}{2}-\dfrac{1}{2} \tanh \eta \right) }{\Gamma (1 \mp is)} \nonumber \\&\qquad \times \left( \dfrac{1+ \tanh \eta }{1- \tanh \eta }\right) ^{\pm is/2}, \end{aligned}$$

where we have exploited the relation ${}_{2}\mathrm {F}_{1}(a,b;c;x)={}_{2}\mathrm {F}_{1}(b,a;c;x)$ to write

138 $$\begin{aligned}&{}_{2}\mathrm {F}_{1}\left( \dfrac{1}{2}-i \vert q\vert ,\dfrac{1}{2}+i \vert q\vert ; 1 \mp is;\dfrac{1}{2}-\dfrac{1}{2} \tanh \eta \right) \nonumber \\&\quad ={}_{2}\mathrm {F}_{1}\left( \dfrac{1}{2}-i q ,\dfrac{1}{2}+i q ; 1 \mp is;\dfrac{1}{2}-\dfrac{1}{2} \tanh \eta \right) . \end{aligned}$$

The expansion about $\eta =0$ of the hypergeometric function occurring above can be written as

139 $$\begin{aligned}&{}_{2}\mathrm {F}_{1}\left( \dfrac{1}{2}-i q ,\dfrac{1}{2}+i q ; 1 \mp is;\dfrac{1}{2}-\dfrac{1}{2} \tanh \eta \right) \nonumber \\&\quad = {}_{2}\mathrm {F}_{1}\left( \dfrac{1}{2}-i q ,\dfrac{1}{2}+i q ; 1 \mp is;\dfrac{1}{2}\right) + \mathrm{O}\left( \eta \right) \nonumber \\&\quad = \dfrac{\sqrt{\pi } \left( 2\right) ^{\pm is} \Gamma \left( 1 \mp is \right) }{\Gamma \left[ \dfrac{1}{2}\left( \dfrac{3}{2}-iq \mp is\right) \right] \Gamma \left[ \dfrac{1}{2}\left( \dfrac{3}{2}+iq \mp is\right) \right] } \nonumber \\&\qquad +\mathrm{O}\left( \eta \right) , \end{aligned}$$

where we have exploited Eq. (15.1.26) in Ref. [53]. Therefore, from Eqs. (137) and (139), we find

140 $$\begin{aligned}&\mathsf {P}^{\pm is}_{\nu }\left( \tanh \eta \right) \overset{\eta \rightarrow 0}{\sim } \, e^{\pm i\eta s} \,\nonumber \\&\quad \times \dfrac{\sqrt{\pi } \left( 2\right) ^{\pm is}}{\Gamma \left[ \dfrac{1}{2}\left( \dfrac{3}{2}-iq \mp is\right) \right] \Gamma \left[ \dfrac{1}{2}\left( \dfrac{3}{2}+iq \mp is\right) \right] }. \end{aligned}$$

Thus, from the last relation jointly with Eq. (89), the early-time expression of the eigenfunctions (83) evaluated in the flat regime (86) become141As a consequence of Eqs. (124) and (141), we find that the early-time propagator can be written as142where “ET” means “early-time” propagator. The first term reads as

143 $$\begin{aligned}&\langle \phi (x) \phi ^*(x^\prime )\rangle ^{\mathrm{FR}}_{\mathrm{ET1}}\nonumber \\&=- 8 R^2 \sum _{l,m} Y^m_l(\theta ,\varphi )\left[ Y^m_l(\theta ^\prime ,\varphi ^\prime )\right] ^*\nonumber \\&\quad \times \int ds dq\, \dfrac{j_l \left( q \chi \right) j_l \left( q \chi ^\prime \right) }{q^{2l}} \nonumber \\&\quad \times \dfrac{ s \,q\, \cosh (\pi q) \sinh (\pi q) \left| \Gamma (iq+l+1) \right| ^2}{\sinh (\pi s) \left( s^2 - q^2 + \dfrac{5}{4}-m^2 R^2 + i \varepsilon \right) \left[ \cosh (2 \pi q)+\cosh (2 \pi s)\right] } \nonumber \\&\quad \times \mathrm{Re} \left[ \dfrac{2^{-2is} \; e^{-is \left( \eta + \eta ^\prime \right) }\;\Gamma ^{-1}\left( \dfrac{1}{2}-iq-is\right) \Gamma ^{-1}\left( \dfrac{1}{2}+iq-is\right) }{\Gamma ^2\left( \dfrac{3}{4}-\dfrac{iq}{2}+\dfrac{is}{2}\right) \Gamma ^2\left( \dfrac{3}{4}+\dfrac{iq}{2}+\dfrac{is}{2}\right) } \right] . \end{aligned}$$

After some calculations, the leading contribution becomes

144 $$\begin{aligned}&\langle \phi (x) \phi ^*(x^\prime )\rangle ^{\mathrm{FR}}_{\mathrm{ET1}}\nonumber \\&\quad =- 4 R^2 \sum _{l,m} Y^m_l(\theta ,\varphi )\left[ Y^m_l(\theta ^\prime ,\varphi ^\prime )\right] ^*\nonumber \\&\qquad \times \int ^{+ \infty }_{- \infty } ds\; 2^{2is} \; e^{is \left( \eta + \eta ^\prime \right) } \int ^{+ \infty }_{- \infty } dq\,\nonumber \\&\qquad \times \dfrac{j_l \left( q \chi \right) j_l \left( q \chi ^\prime \right) }{q^{2l}} \dfrac{ q \sinh (\pi q) \left| \Gamma (iq+l+1) \right| ^2}{ \left( s^2 - q^2 + \dfrac{5}{4} -m^2 R^2 + i \varepsilon \right) } \nonumber \\&\qquad \times \dfrac{ s \cosh (\pi q) }{\sinh (\pi s) \left[ \cosh (2 \pi q)+\cosh (2 \pi s)\right] }\nonumber \\&\qquad \times \dfrac{ \Gamma ^{-1}\left( \dfrac{1}{2}+iq+is\right) \Gamma ^{-1}\left( \dfrac{1}{2}-iq+is\right) }{\Gamma ^2\left( \dfrac{3}{4}+\dfrac{iq}{2}-\dfrac{is}{2}\right) \Gamma ^2\left( \dfrac{3}{4}-\dfrac{iq}{2}-\dfrac{is}{2}\right) } \nonumber \\&=- 8 \pi R^2 \sum _{l,m} Y^m_l(\theta ,\varphi )\left[ Y^m_l(\theta ^\prime ,\varphi ^\prime )\right] ^*\nonumber \\&\qquad \times \int ^{+ \infty }_{- \infty } ds\; 2^{2is} \; e^{is \left( \eta + \eta ^\prime \right) } \nonumber \\&\qquad \times \int ^{+ \infty }_{0} dq\, \dfrac{ q^2 j_l \left( q \chi \right) j_l \left( q \chi ^\prime \right) }{ \left( s^2 - q^2 + \dfrac{5}{4} -m^2 R^2 + i \varepsilon \right) }\nonumber \\&\quad \times \dfrac{ s \cosh (\pi q) }{\sinh (\pi s) \left[ \cosh (2 \pi q)+\cosh (2 \pi s)\right] } \nonumber \\&\qquad \times \dfrac{ \Gamma ^{-1}\left( \dfrac{1}{2}+iq+is\right) \Gamma ^{-1}\left( \dfrac{1}{2}-iq+is\right) }{\Gamma ^2\left( \dfrac{3}{4}+\dfrac{iq}{2}-\dfrac{is}{2}\right) \Gamma ^2\left( \dfrac{3}{4}-\dfrac{iq}{2}-\dfrac{is}{2}\right) }, \end{aligned}$$

where we have exploited Eq. (128) and the fact that the integrand is an even function of *q*. If we exploit the following relations [53]

145 $$\begin{aligned} \Gamma (2z)&= \dfrac{2^{2z-1}}{\sqrt{\pi }} \Gamma (z) \Gamma (z+1/2), \nonumber \\ \Gamma \left( 1/4+iy\right) \Gamma \left( 3/4-i y\right)&=\frac{\pi \sqrt{2}}{\cosh \left( \pi y\right) +i\sinh \left( \pi y \right) }, \end{aligned}$$

then we can simplify the last term occurring in Eq. (144) as

146 $$\begin{aligned}&\dfrac{ \Gamma ^{-1}\left( \dfrac{1}{2}+iq+is\right) \Gamma ^{-1}\left( \dfrac{1}{2}-iq+is\right) }{\Gamma ^2\left( \dfrac{3}{4}+\dfrac{iq}{2}-\dfrac{is}{2}\right) \Gamma ^2\left( \dfrac{3}{4}-\dfrac{iq}{2}-\dfrac{is}{2}\right) }\nonumber \\&\quad = \dfrac{\cosh (\pi q) + i \sinh (\pi s)}{\pi \left( 2^{2is}\right) \left| \Gamma \left( \dfrac{3}{4}+\dfrac{iq}{2}+\dfrac{is}{2}\right) \Gamma \left( \dfrac{3}{4}+\dfrac{iq}{2}-\dfrac{is}{2}\right) \right| ^2}, \end{aligned}$$

and hence, after some algebra, we get

147 $$\begin{aligned}&\langle \phi (x) \phi ^*(x^\prime )\rangle ^{\mathrm{FR}}_{\mathrm{ET1}}\nonumber \\&=- 4 R^2 \sum _{l,m} Y^m_l(\theta ,\varphi )\left[ Y^m_l(\theta ^\prime ,\varphi ^\prime )\right] ^*\nonumber \\&\quad \times \int \limits ^{+ \infty }_{- \infty } ds \int \limits ^{+ \infty }_{0} dq\, \dfrac{ q^2 j_l \left( q \chi \right) j_l \left( q \chi ^\prime \right) e^{is \left( \eta + \eta ^\prime \right) } }{ \left( s^2 - q^2 + \dfrac{5}{4} -m^2 R^2 + i \varepsilon \right) } \nonumber \\&\quad \times \dfrac{ s \cosh (\pi q) }{\sinh (\pi s) \left[ \cosh ( \pi q)-i\sinh ( \pi s)\right] }\nonumber \\&\quad \times \dfrac{1}{\left| \Gamma \left( \dfrac{3}{4}+\dfrac{iq}{2}+\dfrac{is}{2}\right) \Gamma \left( \dfrac{3}{4}+\dfrac{iq}{2}-\dfrac{is}{2}\right) \right| ^2 }. \end{aligned}$$

We can obtain an estimate for the last term appearing in the above equation. Indeed, taking into account the relation [59]

148 $$\begin{aligned} \left| \Gamma (x+iy) \right| ^2 \geqslant \dfrac{\Gamma ^2(x)}{\cosh (\pi y)}, \end{aligned}$$

we obtain the inequality

149 $$\begin{aligned}&\dfrac{1}{\left| \Gamma \left( \dfrac{3}{4}+\dfrac{iq}{2}+\dfrac{is}{2}\right) \Gamma \left( \dfrac{3}{4}+\dfrac{iq}{2}-\dfrac{is}{2}\right) \right| ^2 } \nonumber \\&\quad \leqslant \dfrac{1}{2\, \Gamma ^{4}\left( \dfrac{3}{4}\right) } \left[ \cosh (\pi q)+ \cosh (\pi s)\right] , \end{aligned}$$

where $$\Gamma ^4\left( \tfrac{3}{4}\right) \approx 2.25$$.

The second term appearing in Eq. (142) is

150 $$\begin{aligned}&\langle \phi (x) \phi ^*(x^\prime )\rangle ^{\mathrm{FR}}_\mathrm{ET2}\nonumber \\&=\dfrac{8R^2}{\pi }\sum _{l,m} Y^m_l(\theta ,\varphi )\left[ Y^m_l(\theta ^\prime ,\varphi ^\prime )\right] ^* \nonumber \\&\quad \times \int ds dq \, \dfrac{j_l \left( q \chi \right) j_l \left( q \chi ^\prime \right) }{q^{2l}}\nonumber \\&\quad \times \dfrac{\cos \left[ s \left( \eta -\eta ^\prime \right) \right] \, q \sinh (\pi q) \left| \Gamma (iq+l+1)\right| ^2}{ \left( s^2 - q^2 + \dfrac{5}{4}-m^2 R^2 + i \varepsilon \right) } \nonumber \\&\quad \times \dfrac{s \left[ \sinh ^2( \pi s)+\cosh ^2( \pi q)\right] }{\sinh (\pi s)\left[ \cosh (2 \pi q)+\cosh (2 \pi s)\right] }\nonumber \\&\quad \times \dfrac{1}{\left| \Gamma \left( \dfrac{3}{4}+\dfrac{iq}{2}+\dfrac{is}{2}\right) \Gamma \left( \dfrac{3}{4}+\dfrac{iq}{2}-\dfrac{is}{2}\right) \right| ^2 }, \end{aligned}$$

and, upon taking into account Eq. (128) jointly with the fact that the integrand is an even function of *q*, we obtain, after some algebra,

151 $$\begin{aligned}&\langle \phi (x) \phi ^*(x^\prime )\rangle ^{\mathrm{FR}}_\mathrm{ET2}=4R^2\sum _{l,m} Y^m_l(\theta ,\varphi )\left[ Y^m_l(\theta ^\prime ,\varphi ^\prime )\right] ^*\nonumber \\&\quad \times \int \limits ^{+ \infty }_{- \infty } ds \int \limits ^{+ \infty }_{0} dq\, \dfrac{ q^2 j_l \left( q \chi \right) j_l \left( q \chi ^\prime \right) e^{is \left( \eta - \eta ^\prime \right) } }{ \left( s^2 - q^2 + \dfrac{5}{4} -m^2 R^2 + i \varepsilon \right) } \nonumber \\&\quad \times \dfrac{s}{\sinh (\pi s)} \dfrac{1}{\left| \Gamma \left( \dfrac{3}{4}+\dfrac{iq}{2}+\dfrac{is}{2}\right) \Gamma \left( \dfrac{3}{4}+\dfrac{iq}{2}-\dfrac{is}{2}\right) \right| ^2 }. \end{aligned}$$

Therefore, if we sum the contributions (147) and (151), the early-time propagator (142) becomes152The last term occurring in the above equation can be arranged as follows

153 $$\begin{aligned}&\frac{s}{\sinh (\pi s)} \left[ e^{i s(\eta -\eta ^\prime )} - \dfrac{\cosh (\pi q) e^{i s(\eta +\eta ^\prime )}}{ \cosh ( \pi q)-i\sinh ( \pi s)} \right] \nonumber \\&=\frac{s}{\sinh (\pi s)} \dfrac{\left[ e^{i s(\eta -\eta ^\prime )} - e^{i s(\eta +\eta ^\prime )} \right] \cosh (\pi q) -i \,e^{i s(\eta -\eta ^\prime )} \sinh (\pi s)}{ \cosh ( \pi q)-i\sinh ( \pi s)} \nonumber \\&\overset{\eta ,\eta ^\prime \rightarrow 0}{\sim } \, \dfrac{ -is }{\left[ \cosh ( \pi q)-i\sinh ( \pi s)\right] } e^{i s (\eta - \eta ')}, \end{aligned}$$

and hence, for $\eta , \eta ^\prime \rightarrow 0$, we get the final formula154The early-time propagator (154) turns out to be bounded and well-defined owing to the upper bound (149). It gives rise to a more-or-less standard correlation between points $x,x^\prime$ lying on the same sheet of the projected spacetime $\mathcal{M}^{3,1}$. More remarkably, it also gives rise to a well-defined correlation and in fact a propagation between two points located on opposite sheets of $\mathcal{M}^{3,1}$ near the BB; for larger $|\eta |$ and $|\eta ^\prime |$ this is suppressed compared to the case of two points on the same sheet, due to the oscillating term $e^{is \left( \eta - \eta ^\prime \right) }$.

The observation that a scalar particle can cross the BB agrees at least qualitatively with the classical analysis regarding null and timelike geodesics of Sect. 3.2. A more detailed analysis of this fascinating result is left for future work.

## Conclusions

In this paper, we elaborated in detail the scalar modes and the propagator on a quantum version of a 3 + 1-dimensional FLRW spacetime with BB, in the semi-classical limit. The underlying framework of matrix models provides a clean setup to work with a spacetime which is singular as a classical manifold, but well-defined as a quantum geometry.

The most interesting conclusion is that the physics of scalar fluctuations is perfectly well-defined even at or near the classical singularity, and it is possible to relate the pre- and post-BB eras in a meaningful way. This implies some intriguing correlation between the two sides of the BB, which remain to be worked out in detail. The framework of matrix models provides a clear prescription how to define and compute the propagator, and the causal structure of a Feynman propagator is obtained automatically, at least at late times. This is remarkable, since time emerges on the same footing as space from the underlying matrix model, and there is no a priori notion of classical time.

This work should be seen in the context of emergent spacetime and gravity within the IKKT matrix model, which is closely related to string theory [33]. The present background under considerations then arises as a classical solution [7], and a gravitational action arises from one-loop effects [36], cf. Ref. [37]. Fluctuations are governed by a noncommutative field theory, which for scalar fields reduces to the one elaborated here. This suggests that analogous properties can be expected for all fluctuations in the model. In particular, the resulting theory is expected to satisfy the standard causality and unitarity requirements in quantum field theory, given the emergence of a Feynman propagator and the maximal supersymmetry of the underlying IKKT model. Even though many aspects of such a theory remain to be understood, the results of the present paper should provide sufficient motivation for further studies.

The present analysis is restricted to non-interacting test particles on the background geometry. This is of course not entirely satisfactory, since the density of matter is expected to be singular at the BB, which would lead to modifications of the background and hence of the metric. The inclusion of such effects as well as the induced Einstein–Hilbert action would be very desirable, but is beyond the scope of this paper.

Finally, it is interesting to point out that recent numerical simulations of the bosonic IKKT model with mass term produced evidence for an emergent spacetime, with features reminiscent of a bouncing 1 + 1-dimensional cosmology [60]. One may therefore hope to eventually relate the matrix background under consideration to numerical simulations of the model.

## Data Availability

This manuscript has no associated data or the data will not be deposited. [Authors’ comment: Data sharing is not applicable to this article as no new data were created or analyzed in this study.]

## Appendix A: Eigenfunctions of Laplacian operators

In this appendix, we derive the eigenfunctions of the operators $\Delta _{\mathscr {G}}$ (see Sect. A.1) and $\Delta ^{(3)}$ (see Sect. A.2). For our convenience, we report their expressions also here:

A.1 $$\begin{aligned} \Delta _{\mathscr {G}} \phi&= \dfrac{1}{R^2}\Biggl \{ 3 \tanh (\eta ) \partial _\eta + \partial ^2_\eta +\dfrac{1}{\cosh ^2 (\eta )} \nonumber \\&\quad \times \Biggl [ \dfrac{2}{\tanh (\chi )}\partial _\chi +\partial ^2_\chi +\dfrac{1}{\sinh ^2(\chi )}\nonumber \\&\quad \times \left( \dfrac{1}{\tan (\theta )}\partial _\theta +\partial ^2_\theta +\dfrac{1}{\sin ^2(\theta ) } \partial ^2_\varphi \right) \Biggr ]\Biggr \}\phi , \end{aligned}$$

A.2 $$\begin{aligned} \Delta ^{(3)} \phi&= \Biggl [ \left( \dfrac{2}{\tanh \chi }\partial _\chi + \partial ^2_\chi \right) + \dfrac{1}{\sinh ^2 \chi } \nonumber \\&\quad \times \left( \dfrac{1}{\tan \theta } \partial _\theta + \partial ^2_\theta + \dfrac{1}{\sin ^2 \theta }\partial ^2_\varphi \right) \Biggr ]\phi . \end{aligned}$$

### A.1: Eigenfunctions of the Laplacian operator $\Delta _{\mathscr {G}}$

In this section, we solve the equation

A.3 $$\begin{aligned} \Delta _{\mathscr {G}} \phi = \lambda \phi , \end{aligned}$$

defining the eigenfunctions of the Laplacian operator (A.1). By exploiting the separation *ansatz*

A.4 $$\begin{aligned} \phi (\eta ,\chi ,\theta ,\varphi )&= \hat{f}(\eta )\hat{g}(\chi )Y^m_l(\theta ,\varphi ), \end{aligned}$$

Equations (A.3) gives

A.5 $$\begin{aligned}&\dfrac{1}{\hat{f}}\left( 3 \tanh (\eta ) \partial _\eta + \partial ^2_\eta \right) \hat{f} +\dfrac{1}{\hat{g}}\dfrac{1}{\cosh ^2 \eta } \nonumber \\&\quad \times \left( \dfrac{2}{\tanh \chi }\partial _\chi +\partial ^2_\chi \right) \hat{g} -\dfrac{l(l+1)}{\cosh ^2 \eta \sinh ^2 \chi } = \lambda R^2, \end{aligned}$$

where we have exploited that the spherical harmonic functions $Y^m_l(\theta ,\varphi )$ satisfy the property (56). By means of the method of separation of variables, the above equation leads to the ordinary differential equations

A.6a $$\begin{aligned} \left( \partial ^2_\eta + 3 \tanh (\eta ) \partial _\eta -\lambda R^2 -\dfrac{\varrho }{\cosh ^2 \eta } \right) \hat{f}(\eta )&=0, \end{aligned}$$

A.6b $$\begin{aligned} \left( \partial ^2_\chi +\dfrac{2}{\tanh \chi } \partial _\chi -\dfrac{l(l+1)}{\sinh ^2 \chi } + \varrho \right) \hat{g}(\chi )&=0, \end{aligned}$$

where $\varrho$ is a real constant.

Equation (A.6a) can be put in the form of a general Legendre equation [53]. Indeed, following the line of reasoning of Sect. 4.2, we introduce the variable

A.7 $$\begin{aligned} z=\tanh \eta \in (-1,1), \end{aligned}$$

and write the derivative operator $\partial _\eta$ as

A.8 $$\begin{aligned} \partial _\eta&= (1-z^2)\partial _z. \end{aligned}$$

In this way, Eq. (A.6a) becomes

A.9 $$\begin{aligned} \left[ (1-z^2) \partial ^2_z + z \partial _z - \varrho -\dfrac{\lambda R^2}{1-z^2} \right] \hat{f}(z)=0, \end{aligned}$$

which, upon introducing

A.10 $$\begin{aligned} \hat{f}(z)=(1-z^2)^{3/4}\hat{h}(z), \end{aligned}$$

amounts to the general Legendre equation

A.11 $$\begin{aligned} (1-z^2)\partial ^2_z&\hat{h} -2z\partial _z \hat{h} + \left[ \left( \dfrac{3}{4}-\varrho \right) -\left( \dfrac{\dfrac{9}{4} +\lambda R^2}{1-z^2}\right) \right] \nonumber \\&\quad \times \hat{h}=0. \end{aligned}$$

This equation can be solved via the associated Legendre functions of the first and second kind $\mathsf {P}^{\hat{\mu }}_{\hat{\nu }}(z)$ and $\mathsf {Q}^{\hat{\mu }}_{\hat{\nu }}(z)$, respectively, having degree $\hat{\nu }$ and order $\hat{\mu }$ given by

A.12a $$\begin{aligned} \hat{\nu }&= \dfrac{1}{2}\left( 2\sqrt{1-\varrho }-1\right) , \end{aligned}$$

A.12b $$\begin{aligned} \hat{\mu }&= \dfrac{1}{2}\sqrt{9+4R^2\lambda }. \end{aligned}$$

Therefore, the solution of Eq. (A.6a) is (cf. Eqs. (A.7) and (A.10))

A.13 $$\begin{aligned} \hat{f}(\eta )= (1-\tanh ^2 \eta )^{3/4}\left[ c_1 \mathsf {P}^{\hat{\mu }}_{\hat{\nu }}(\tanh \eta ) +c_2 \mathsf {Q}^{\hat{\mu }}_{\hat{\nu }}(\tanh \eta )\right] , \end{aligned}$$

$c_1$ and $c_2$ being integration constants.

The solution of (A.6b) can be written in terms of the ordinary hypergeometric function [53] as

A.14 $$\begin{aligned} \hat{g}(\chi )&= \dfrac{\left( 1-\tanh ^2 \chi \right) ^{(1/2)(1-a+\hat{b}+\hat{c})}}{\left( \tanh \chi \right) ^{3/2}}\nonumber \\&\quad \times \Biggl [ c_3 \left( \tanh ^2 \chi \right) ^{a/2} {}_{2}\mathrm {F}_{1}\left( \hat{b},\hat{c};a;\tanh ^2 \chi \right) \nonumber \\&\quad + c_4 \left( -1\right) ^{1-a}\left( \tanh ^2 \chi \right) ^{1-a/2}\nonumber \\&\quad \times {}_{2}\mathrm {F}_{1}\left( 1-a+\hat{b},1-a+\hat{c};2-a;\tanh ^2 \chi \right) \Biggr ], \end{aligned}$$

where $c_3$ and $c_4$ are integration constants and

A.15 $$\begin{aligned} a&\equiv \dfrac{1}{2} \left( 3+2l\right) , \nonumber \\ \hat{b}&\equiv \dfrac{1}{2} \left( 1+l+\sqrt{1-\varrho }\right) , \nonumber \\ \hat{c}&\equiv \dfrac{1}{2} \left( 2+l+\sqrt{1-\varrho }\right) = \hat{b} + \dfrac{1}{2}. \end{aligned}$$

We can exploit the same approach as in Sect. 4.2 and write Eq. (A.14) in a different form. Indeed, if we introduce the variable

A.16 $$\begin{aligned} y=\coth \chi , \end{aligned}$$

then Eq. (A.6b) can be written as (cf. Eq. (69))

A.17 $$\begin{aligned} (1-y^2)\partial ^2_y \hat{g}(y)+ \left[ l(l+1)+\dfrac{\varrho }{1-y^2}\right] \hat{g}(y)=0, \end{aligned}$$

and admits the solution

A.18 $$\begin{aligned} \hat{g}(\chi )&= \sqrt{\coth ^2 \chi -1} \left[ c_3 \mathcal {P}^{\sqrt{1-\varrho }}_l(\coth \chi ) \right. \nonumber \\&\quad \left. +c_4 \mathcal {Q}^{\sqrt{1-\varrho }}_l(\coth \chi ) \right] , \end{aligned}$$

where we have exploited the substitution (A.16).

Therefore, the eigenfunctions (A.4) of the Laplacian operator (A.1) can be expressed in terms the solutions (A.13) and (A.18). Furthermore, from Eq. (A.12b) we obtain the explicit expression of the eigenvalue $\lambda$, i.e.,

A.19 $$\begin{aligned} \lambda = \dfrac{4 \hat{\mu }^2 -9}{4R^2}. \end{aligned}$$

It is worth underling the similarities between the eigenfunctions (A.13), (A.18) of $\Delta _{\mathscr {G}}$ and (65), (71) of the d’Alembertian operator $\Box$. The only differences between the two sets of solutions involve the degrees and the orders of the underlying associated Legendre functions (cf. Eqs. (A.12) and (64)). These features stem from the relation (52).

### A.2: Eigenfunctions of the Laplacian operator $\Delta ^{(3)}$

In this section, we derive the eigenfunctions of the Laplacian operator (A.2). Like in the previous section, we solve the equation

A.20 $$\begin{aligned} \Delta ^{(3)} \phi (\chi ,\theta ,\varphi )=\lambda \phi (\chi ,\theta ,\varphi ), \end{aligned}$$

by means of the separation *ansatz*

A.21 $$\begin{aligned} \phi (\chi ,\theta ,\varphi ) = \tilde{g}(\chi ) Y^m_l(\theta ,\varphi ), \end{aligned}$$

$Y^m_l(\theta ,\varphi )$ being the spherical harmonic function. In this way, after a straightforward computation, from Eq. (A.20) we obtain

A.22 $$\begin{aligned} \left[ \partial ^2_\chi +\dfrac{2}{\tanh \chi }\partial _\chi - \dfrac{l(l+1)}{\sinh ^2 \chi }-\lambda \right] \tilde{g}(\chi )=0, \end{aligned}$$

where we have exploited Eq. (56). The solution of the differential equation (A.22) reads as

A.23 $$\begin{aligned} \tilde{g}(\chi )&= \dfrac{\left( 1-\tanh ^2 \chi \right) ^{(1/2)(1-a+\tilde{b}+\tilde{c})}}{\left( \tanh \chi \right) ^{3/2}}\nonumber \\&\quad \times \Biggl [ c_1 \left( \tanh ^2 \chi \right) ^{a/2} {}_{2}\mathrm {F}_{1}\left( \tilde{b},\tilde{c};a;\tanh ^2 \chi \right) \nonumber \\&\quad + c_2 \left( -1\right) ^{1-a}\left( \tanh ^2 \chi \right) ^{1-a/2}\nonumber \\&\quad \times {}_{2}\mathrm {F}_{1}\left( 1-a+\tilde{b},1-a+\tilde{c};2-a;\tanh ^2 \chi \right) \Biggr ], \end{aligned}$$

where $c_1$, $c_2$ denote the integration constants and

A.24 $$\begin{aligned} a&\equiv \dfrac{1}{2} \left( 3+2l\right) , \nonumber \\ \tilde{b}&\equiv \dfrac{1}{2} \left( 1+l+\sqrt{1+\lambda }\right) , \nonumber \\ \tilde{c}&\equiv \dfrac{1}{2} \left( 2+l+\sqrt{1+\lambda }\right) = \tilde{b} + \dfrac{1}{2}. \end{aligned}$$

In the same way as in Sects. 4.2 and A.1, we can write the solution of (A.22) in terms of the associated Legendre functions. Indeed, by introducing

A.25 $$\begin{aligned} y=\coth \chi , \end{aligned}$$

and bearing in mind Eq. (69), we end up with

A.26 $$\begin{aligned} \tilde{g}(\chi )&=\sqrt{\coth ^2 \chi -1} \left[ c_1 \mathcal {P}^{\sqrt{1+\lambda }}_l(\coth \chi ) \right. \nonumber \\&\quad \left. +c_2 \mathcal {Q}^{\sqrt{1+\lambda }}_l(\coth \chi ) \right] . \end{aligned}$$

Note that, owing to Eq. (50), Eqs. (A.18) and (A.26) have the same structure (the only difference consists in the orders of the Legendre functions).

## Appendix B: Orthogonality relations of the associated Legendre functions

In this appendix, we will provide the details of the calculations leading to the results (97) and (105).

We will adopt the following conventions:

- $\mathsf {P}^{\mu }_\nu (x), \mathsf {Q}^{\mu }_\nu (x)$ denote the associated Legendre functions of the first and second kind, respectively, with *x* lying in the interval $(-1,1)$;
- $\mathcal {P}^{\mu }_\nu (x), \mathcal {Q}^{\mu }_\nu (x)$ denote the associated Legendre functions of the first and second kind, respectively, with *x* lying in the interval $(1,+\infty )$.

In Ref. [56], it has been shown that

B.1 $$\begin{aligned}&\int ^1_{-1} \dfrac{dx}{1-x^2}\mathsf {P}^{is^\prime }_\nu (x) \mathsf {P}^{is}_\nu (x) = -\dfrac{2 \Gamma (is)\Gamma (-is)}{\Gamma (1+\nu -is)\Gamma (-\nu -is)}\nonumber \\&\quad \times \sin (\pi \nu )\delta (s-s^\prime ) \nonumber \\&\quad +\Biggl [\dfrac{\pi }{\Gamma (1-is)\Gamma (1+is)}+\dfrac{\sin ^2(\pi \nu )\Gamma (is)\Gamma (-is)}{\pi } \nonumber \\&\quad + \dfrac{\pi \Gamma (is)\Gamma (-is) }{\Gamma (1+\nu -is)\Gamma (-\nu -is) \Gamma (1+\nu +is)\Gamma (-\nu +is)}\Biggr ]\nonumber \\&\quad \times \delta (s+s^\prime ), \end{aligned}$$

which, upon exploiting the relations [53]

B.2a $$\begin{aligned} \Gamma (is)\Gamma (-is)&= \dfrac{\pi }{s \sinh (\pi s)}, \end{aligned}$$

B.2b $$\begin{aligned} \Gamma (1-is)\Gamma (1+is)&= \dfrac{\pi s}{\sinh (\pi s)}, \end{aligned}$$

B.2c $$\begin{aligned} \Gamma (1+\nu -is)\Gamma (-\nu +is)&= \dfrac{\pi }{\sin \left[ \pi (-\nu +i s)\right] }, \end{aligned}$$

B.2d $$\begin{aligned} \Gamma (1+\nu +is)\Gamma (-\nu -is)&= \dfrac{\pi }{\sin \left[ \pi (-\nu -i s)\right] }, \end{aligned}$$

can be simplified, yielding

B.3 $$\begin{aligned}&\int ^1_{-1} \dfrac{dx}{1-x^2}\mathsf {P}^{is^\prime }_\nu (x) \mathsf {P}^{is}_\nu (x) \nonumber \\&\quad = -\dfrac{2 \pi \sin (\pi \nu )}{s \sinh (\pi s)}\dfrac{1}{\Gamma (1+\nu -is)\Gamma (-\nu -is)}\delta (s-s^\prime ) \nonumber \\&\qquad +2\left[ \dfrac{\sinh (\pi s)}{s}+\dfrac{\sin ^2(\pi \nu )}{s \sinh (\pi s)}\right] \delta (s+s^\prime ). \end{aligned}$$

Following the same strategy as in Ref. [56], we will now show that

B.4 $$\begin{aligned} \int ^{+\infty }_{1} \dfrac{dx}{1-x^2}\mathcal {Q}^{iq^\prime }_\nu (x) \mathcal {Q}^{iq}_\nu (x)= -\dfrac{\left( \pi /2\right) ^2}{q \sinh \left( \pi q\right) } \delta (q+q^\prime ), \end{aligned}$$

provided $$\nu >-3$$.

The explicit form of $\mathcal {Q}^{\mu }_\nu (x)$ reads either as [55]

B.5 $$\begin{aligned} \mathcal {Q}^{\mu }_{\nu }\left( x\right)&=\frac{e^{i \pi \mu }\sqrt{\pi }\Gamma \left( \nu +\mu +1 \right) \left( x^{2}-1\right) ^{\mu /2}}{2^{\nu +1}x^{\nu +\mu +1}}\nonumber \\&\quad \times \mathbf {F}\left( \tfrac{1}{2}\nu +\tfrac{1}{2}\mu +1,\tfrac{1}{2}\nu +\tfrac{1}{2}\mu +\tfrac{1}{2} ;\nu +\tfrac{3}{2};\frac{1}{x^{2}}\right) , \end{aligned}$$

or

B.6 $$\begin{aligned} \mathcal {Q}^{\mu }_{\nu }\left( x\right)&=\frac{2^{\nu } e^{i \pi \mu }\Gamma \left( \nu +1\right) \Gamma \left( \nu +\mu +1\right) (x+1)^{\mu /2}}{(x-1)^{(\mu /2)+\nu +1}}\nonumber \\&\quad \times \mathbf {F}\left( \nu +1,\nu +\mu +1;2\nu +2; \frac{2}{1-x}\right) , \end{aligned}$$

where $\mathbf {F}\left( a,b;c;x\right)$ denotes the scaled (or Olver’s) hypergeometric function, which is defined by

B.7 $$\begin{aligned} \mathbf {F}\left( a,b;c;x\right)&= \dfrac{1}{\Gamma (a)\Gamma (b)} \sum _{n=0}^{+\infty }\dfrac{\Gamma (a+n)\Gamma (b+n)}{\Gamma (c+n)}\dfrac{x^n}{n!}\nonumber \\&= \dfrac{{}_{2}\mathrm {F}_{1}\left( a,b;c;x\right) }{\Gamma (c)}, \end{aligned}$$

${}_{2}\mathrm {F}_{1}\left( a,b;c;x\right)$ being the hypergeometric function. Starting from Eqs. (B.5) and (B.6), one finds the following asymptotic approximations [55]:

B.8 $$\begin{aligned} \mathcal {Q}^\mu _\nu (x)&\overset{x \rightarrow + \infty }{\sim } \dfrac{e^{i \pi \mu } \sqrt{\pi }}{2^{\nu +1}} \dfrac{\Gamma (\nu + \mu + 1)}{\Gamma (\nu + 3/2)} \dfrac{1}{x^{\nu +1}} + \mathrm{O}\left( x^{-\nu -3}\right) , \end{aligned}$$

B.9 $$\begin{aligned} \mathcal {Q}^\mu _\nu (x)&\overset{x \rightarrow 1^{+}}{\sim } \dfrac{e^{i \pi \mu }}{2} \Gamma (\mu ) \left( \dfrac{x+1}{x-1}\right) ^{\mu /2} \left[ 1+\mathrm{O}\left( x-1\right) \right] . \end{aligned}$$

Given the above premises, we can now prove Eq. (B.4). First of all, the general Legendre differential equation [53]

B.10 $$\begin{aligned} \dfrac{d}{dx}\left[ (1-x^2)\dfrac{df(x)}{dx}\right] + \left[ \nu (\nu +1) -\dfrac{\mu ^2}{1-x^2}\right] f(x)=0, \end{aligned}$$

written for the associated Legendre functions $\mathcal {Q}^{iq}_{\nu }(x)$ and $\mathcal {Q}^{iq^\prime }_{\nu }(x)$ having a purely imaginary order yields

B.11 $$\begin{aligned}&\dfrac{d}{dx}\left[ (1-x^2)\dfrac{d\mathcal {Q}^{iq}_{\nu }(x)}{dx}\right] \nonumber \\&\quad + \left[ \nu (\nu +1) +\dfrac{q^2}{1-x^2}\right] \mathcal {Q}^{iq}_{\nu }(x)=0, \end{aligned}$$

and

B.12 $$\begin{aligned}&\dfrac{d}{dx}\left[ (1-x^2)\dfrac{d\mathcal {Q}^{iq^\prime }_{\nu }(x)}{dx}\right] \nonumber \\&\quad + \left[ \nu (\nu +1) +\dfrac{q^{\prime 2}}{1-x^2}\right] \mathcal {Q}^{iq^\prime }_{\nu }(x)=0, \end{aligned}$$

respectively. If we subtract Eq. (B.12) multiplied by $\mathcal {Q}^{iq}_{\nu }(x)$ from Eq. (B.11) multiplied by $\mathcal {Q}^{iq^\prime }_{\nu }(x)$, we find, after integrating by part,

B.13 $$\begin{aligned}&\int ^\zeta _\xi \dfrac{dx}{1-x^2}\mathcal {Q}^{iq}_{\nu }(x)\mathcal {Q}^{iq^\prime }_{\nu }(x) = \dfrac{1}{q^{\prime 2}-q^2} \left\{ (1-x^2)\right. \nonumber \\&\quad \times \left. \left[ \mathcal {Q}^{iq^\prime }_{\nu }(x) \dfrac{d}{dx} \mathcal {Q}^{iq}_{\nu }(x) - \mathcal {Q}^{iq}_{\nu }(x) \dfrac{d}{dx} \mathcal {Q}^{iq^\prime }_{\nu }(x) \right] \right\} ^{x=\zeta }_{x=\xi }, \end{aligned}$$

where $\xi ,\zeta \in (1,+\infty )$, with $\xi <\zeta$. Therefore,

B.14 $$\begin{aligned}&\int ^{+\infty }_1 \dfrac{dx}{1-x^2}\mathcal {Q}^{iq}_{\nu }(x)\mathcal {Q}^{iq^\prime }_{\nu }(x)\nonumber \\&= \lim _{\zeta \rightarrow + \infty } \dfrac{1-\zeta ^2}{q^{\prime 2}-q^2}\nonumber \\&\quad \times \left[ \mathcal {Q}^{iq^\prime }_{\nu }(\zeta ) \dfrac{d}{d \zeta } \mathcal {Q}^{iq}_{\nu }(\zeta ) - \mathcal {Q}^{iq}_{\nu }(\zeta ) \dfrac{d}{d \zeta } \mathcal {Q}^{iq^\prime }_{\nu }(\zeta ) \right] \nonumber \\&\quad - \lim _{\xi \rightarrow 1^+} \dfrac{1-\xi ^2}{q^{\prime 2}-q^2} \left[ \mathcal {Q}^{iq^\prime }_{\nu }(\xi ) \dfrac{d}{d \xi } \mathcal {Q}^{iq}_{\nu }(\xi ) \right. \nonumber \\&\quad \left. - \mathcal {Q}^{iq}_{\nu }(\xi ) \dfrac{d}{d \xi } \mathcal {Q}^{iq^\prime }_{\nu }(\xi ) \right] . \end{aligned}$$

The limit involving the $\zeta$ variable can be easily evaluated. Indeed, it follows from Eq. (B.8) that

B.15 $$\begin{aligned}&\lim _{\zeta \rightarrow + \infty } \dfrac{1-\zeta ^2}{q^{\prime 2}-q^2} \left[ \mathcal {Q}^{iq^\prime }_{\nu }(\zeta ) \dfrac{d}{d \zeta } \mathcal {Q}^{iq}_{\nu }(\zeta ) - \mathcal {Q}^{iq}_{\nu }(\zeta ) \dfrac{d}{d \zeta } \mathcal {Q}^{iq^\prime }_{\nu }(\zeta ) \right] \nonumber \\&= \lim _{\zeta \rightarrow + \infty } \dfrac{1-\zeta ^2}{q^{\prime 2}-q^2} \left[ 0 + \mathrm{O}\left( \zeta ^{-\nu -5}\right) \right] =0 , \qquad \nonumber \\&\quad (\text {if }\nu >-3). \end{aligned}$$

For the second limit occurring in Eq. (B.14), we have, after having employed Eq. (B.9),

B.16 $$\begin{aligned}&- \lim _{\xi \rightarrow 1^+} \dfrac{1-\xi ^2}{q^{\prime 2}-q^2} \left[ \mathcal {Q}^{iq^\prime }_{\nu }(\xi ) \dfrac{d}{d \xi } \mathcal {Q}^{iq}_{\nu }(\xi ) - \mathcal {Q}^{iq}_{\nu }(\xi ) \dfrac{d}{d \xi } \mathcal {Q}^{iq^\prime }_{\nu }(\xi ) \right] \nonumber \\&\quad = \dfrac{i \Gamma (i q^\prime ) \Gamma (i q)}{4 e^{\pi (q+q^\prime )}} \lim _{\xi \rightarrow 1^+} \dfrac{e^{-(i/2)(q+q^\prime )\log \left[ (\xi -1)/(\xi +1)\right] }}{q+q^\prime }\nonumber \\&\qquad \times \left[ 1+\mathrm{O}\left( \xi -1\right) \right] , \end{aligned}$$

which can be worked out as follows. If we define the variable

B.17 $$\begin{aligned} u= -\dfrac{1}{2} \log \left( \dfrac{\xi -1}{\xi +1}\right) \overset{\xi \rightarrow 1^+ }{\longrightarrow } + \infty , \end{aligned}$$

then we find

B.18 $$\begin{aligned}&\lim _{\xi \rightarrow 1^+} \dfrac{e^{-(i/2)(q+q^\prime )\log \left[ (\xi -1)/(\xi +1)\right] }}{q+q^\prime } \nonumber \\&\quad = \lim _{u \rightarrow +\infty } \dfrac{e^{iu\left( q+q^\prime \right) }}{q+q^\prime } \nonumber \\&\quad = \lim _{u \rightarrow +\infty } \dfrac{\cos \left[ u\left( q+q^\prime \right) \right] }{q+q^\prime } + i \lim _{u \rightarrow +\infty } \dfrac{\sin \left[ u\left( q+q^\prime \right) \right] }{q+q^\prime }. \end{aligned}$$

Now, we exploit that, in the distributional sense,

B.19 $$\begin{aligned} \lim _{u \rightarrow +\infty } \cos (ux)=0; \end{aligned}$$

furthermore,

B.20 $$\begin{aligned} \lim _{u \rightarrow +\infty } \dfrac{\sin (ux)}{x}&= \dfrac{1}{2} \lim _{u \rightarrow +\infty } \dfrac{e^{iux}-e^{-iux}}{ix}\nonumber \\&= \dfrac{1}{2} \lim _{u \rightarrow +\infty } \int _{-u}^{u} e^{ix u^\prime } du^\prime = \pi \delta (x). \end{aligned}$$

Therefore, Eq. (B.18) gives

B.21 $$\begin{aligned}&\lim _{\xi \rightarrow 1^+} \dfrac{e^{-(i/2)(q+q^\prime )\log \left[ (\xi -1)/(\xi +1)\right] }}{q+q^\prime } =i \pi \delta (q+q^\prime ), \end{aligned}$$

and hence, bearing in mind Eqs. (B.14)–(B.16), we find

B.22 $$\begin{aligned}&\int ^{+\infty }_1 \dfrac{dx}{1-x^2}\mathcal {Q}^{iq}_{\nu }(x)\mathcal {Q}^{iq^\prime }_{\nu }(x)\nonumber \\&\quad = -\dfrac{\pi }{4}\dfrac{\Gamma (iq)\Gamma (iq^\prime )}{e^{\pi (q+q^\prime )}}\delta (q+q^{\prime })\nonumber \\&\quad =-\dfrac{\pi }{4}\Gamma (iq)\Gamma (-iq)\delta (q+q^{\prime }), \nonumber \\&\qquad (\text {with }\nu >-3), \end{aligned}$$

which becomes

B.23 $$\begin{aligned}&\int ^{+\infty }_1 \dfrac{dx}{1-x^2}\mathcal {Q}^{iq}_{\nu }(x)\mathcal {Q}^{iq^\prime }_{\nu }(x) = -\dfrac{(\pi /2)^2}{q \sinh (\pi q)}\delta (q+q^{\prime }), \nonumber \\&\quad (\text {with }\nu >-3), \end{aligned}$$

for any real $q,q^{\prime }$, once we have exploited the identity (B.2a).

The results (B.3) and (B.23) have been exploited in the derivation of Eq. (106).

We conclude this section with a last crucial identity regarding the complex conjugation of the associated Legendre functions of second kind $\mathcal {Q}^{iq}_{l}\left( x\right)$ (with $q \in \mathbb {R}$, $l>0$). These functions can also be written via

B.24 $$\begin{aligned} \varvec{Q}^{iq}_{l}\left( x\right) =\frac{\mathcal {Q}^{iq}_{l}\left( x\right) }{e^{ -\pi q}\Gamma \left( iq+l+1\right) }, \end{aligned}$$

where $\varvec{Q}^{iq}_{l}\left( x\right)$ are referred to as Olver’s associated Legendre functions and satisfy [55]

B.25 $$\begin{aligned} \left[ \varvec{Q}^{iq}_{l}\left( x\right) \right] ^* = \varvec{Q}^{-iq}_{l}\left( x\right) . \end{aligned}$$

Starting from the above relations, it is possible to prove that

B.26 $$\begin{aligned} \left[ \mathcal {Q}^{iq}_l(x)\right] ^* = e^{-2 \pi q} \mathcal {Q}^{-iq}_{l}(x). \end{aligned}$$

## Footnotes

1 See also e.g. [27–32] for related work.

2 By “mild singularity” we do not refer to the kind of singularities studied in Refs. [47, 48].

3 We will consider this regime also in the computation of the propagator for $\eta \rightarrow 0$, where the geometry is strictly speaking no longer flat.

4 The expansion of $\mathcal {Q}^{iq}_l(\coth \chi )$ when $\chi \rightarrow 0$ can be read off from Eq. (B.8); for $j_l(q\chi )$ we refer the reader to Ref. [52].

5 Strictly speaking, the underlying space is twistor space ${\mathbb C}P^{1,2}$, and we are restricting ourselves to the lowest sector of scalar modes here [7].

6 Notice that if we would keep here only the asymptotic behavior of the spherical Bessel functions $j_l$ rather than their full form, the propagator would be ill-defined, as the sum over *l*, *m* would lead to a singular dependence on $\theta ,\varphi$.
